# Structure and Function of a Class III Metal-Independent Lanthipeptide Synthetase

**DOI:** 10.1021/acscentsci.3c00484

**Published:** 2023-10-12

**Authors:** Andrea Hernandez Garcia, Satish K. Nair

**Affiliations:** † Department of Biochemistry, University of Illinois at Urbana−Champaign, Roger Adams Laboratory, 600 S. Mathews Ave., Urbana, Illinois 61801, United States; ‡ Center for Biophysics and Computational Biology, University of Illinois at Urbana−Champaign, Roger Adams Laboratory, 600 S. Mathews Ave., Urbana, Illinois 61801, United States; § Carl R. Woese Institute for Genomic Biology, University of Illinois at Urbana−Champaign, 1206 W. Gregory Drive, Urbana, Illinois 61801, United States

## Abstract

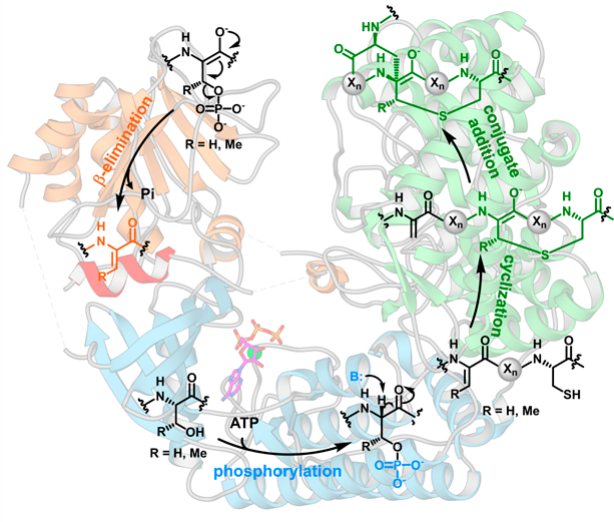

In bacteria, Ser/Thr protein kinase-like sequences are found as part of large multidomain polypeptides that biosynthesize lanthipeptides, a class of natural products distinguished by the presence of thioether cross-links. The kinase domain phosphorylates Ser or Thr residues in the peptide substrates. Subsequent β-elimination by a lyase domain yields electrophilic dehydroamino acids, which can undergo cyclase domain-catalyzed cyclization to yield conformationally restricted, bioactive compounds. Here, we reconstitute the biosynthetic pathway for a class III lanthipeptide from *Bacillus thuringiensis* NRRL B-23139, including characterization of a two-component protease for leader peptide excision. We also describe the first crystal structures of a class III lanthipeptide synthetase, consisting of the lyase, kinase, and cyclase domains, in various states including complexes with its leader peptide and nucleotide. The structure shows interactions between all three domains that result in an active conformation of the kinase domain. Biochemical analysis demonstrates that the three domains undergo movement upon binding of the leader peptide to establish interdomain allosteric interactions that stabilize this active form. These studies inform on the regulatory mechanism of substrate recognition and provide a framework for engineering of variants of biotechnological interest.

## Abstract 2

We utilize a multi-pronged approach to understand the mechanics of lanthipeptide formation by a class III system.

## Introduction

The addition of phosphate groups onto protein or peptide substrates is one of the most widely studied class of post-translational modifications.^1,2^ Such phosphorylation reactions are catalyzed by protein kinases that transfer the γ-phosphate from ATP to principally Ser, Thr, or Tyr residues in the substrate protein.^3,4^ While kinases have historically been most studied in the context of eukaryotic signaling pathways,^5^ recent efforts have identified numerous classes of bacterial kinases that function in prokaryotic regulation, signaling, and biosynthesis.^6,7^ One of the largest classes bacterial protein kinases identified are those that function in the biosynthesis of secondary metabolites such as coenzyme Q, polysaccharide O antigen, and other cell wall components.^8−10^ Other examples of biosynthetic kinases include members of the Aph1 phosphotransferase family (Pfam PF01636)^11^ and polypeptides with protein kinase domain (PF00069) that function in the production of ribosomally synthesized and post-translationally modified peptides (RiPPs).^12,13^

**Figure 1:**
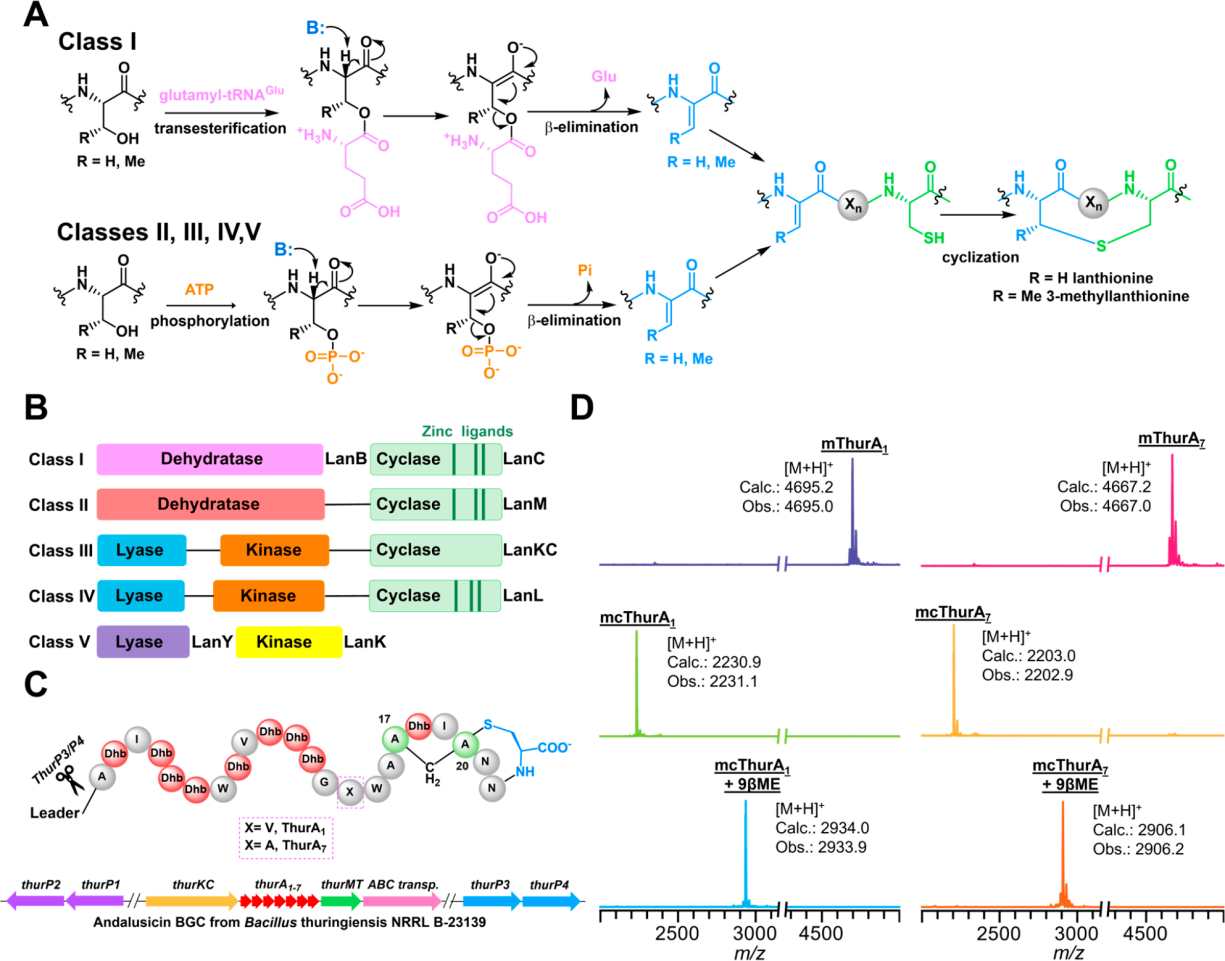
Andalusicin biosynthesis is catalyzed by a class III lanthipeptide synthetase. (A) Mechanisms for various classes of lanthionine synthases. (B) Domain organization of the enzyme(s) that produce lanthipeptides from classes I–V. (C) Biosynthetic gene cluster and external genome proteases that produce andalusicin, including the class III lanthipeptide synthetase ThurKC, precursor peptides ThurA_1–7_, and an α-N methyltransferase ThurMT. The protease complexes ThurP1/P2 and ThurP3/P4 are found outside of the BGC. The expected product following *E. coli* coexpression of ThurKC and ThurA_1_ and ThurA_7_ are also shown. (D) The MALDI-TOF MS of ThurA_1_ (left) and ThurA_7_ (right) coexpressed with ThurKC displays an observed mass corresponding to 11 dehydrations (top). Treatment of the modified peptide with the protease ThurP3/P4 results in leader removal (middle). Treatment of core ThurA_1_ (left) or ThurA_7_ (right) with β-mercaptoethanol shows 9 adducts, consistent with the expected labionin (bottom).

Proteins with kinase-like sequences are especially prevalent in the biosynthesis of lanthipeptides, a class of RiPPs characterized by the presence of lanthionine and/or 3-methyllanthionine rings across the peptide backbone.^14^ In the biosynthesis of lanthipeptides, lanthionine residues are post-translationally introduced into the precursor peptide (generic name LanA) by one of two pathways. In class I systems, a dehydratase (LanB) carries out a glutamyl-tRNA^Glu^-dependent dehydration of Ser/Thr residues in LanA to generate α,β-unsaturated Dha/Dhb residues (Figure 1A).^15,16^ Subsequently, a cyclase (LanC) catalyzes the conjugate addition of a Cys thiolate on to the dehydroamino acid to produce the lanthionine ring (Figure 1A).^17^ Biosynthesis of classes II–IV lanthipeptides requires only a single modification enzyme, which utilizes ATP-dependent kinase-like domains to phosphorylate Ser or Thr and then is eliminated to yield dehydroamino acid, followed by cyclization catalyzed by a cyclase domain.^18^ The class II lanthipeptide synthetases (LanM) have an N-terminal dehydration domain that resembles a lipid kinase (PF13575), where both phosphorylation and β-elimination of phosphate occur, and a C-terminal LanC-like cyclase domain (Figure 1B).^19,20^ Both the class III (LanKC) and class IV (LanL) polypeptides contain a central Ser/Thr kinase-like domain (PF00069) flanked by an N-terminal phospho-Ser/Thr lyase domain and a C-terminal LanC-like cyclase domain.^21−23^ The class V lanthipeptide biosynthetic clusters contain genes with homology to Aph1-like protein kinases (LanK; PF01636) and the type III effector HopA1 (LanY; FP17914).^24−26^

As with all RiPPs, the peptide precursors of lanthipeptides are genetically encoded and consist of an N-terminal leader sequence that recruits the modification enzymes and a C-terminal core where chemistry occurs.^12^ In many RiPP biosynthetic pathways, the leader sequences of peptide substrates are engaged through a small domain that exists either as a stand-along protein^27^ or as a fusion with the catalytic domain of modification enzymes.^28^ These leader-binding domains fall into the PqqD superfamily (IPR00872) and are referred to as RiPP recognition elements (RREs) when present in RiPP biosynthetic clusters.^29^ However, while each of the core biosynthetic enzymes in class II–V lanthipeptide clusters can bind to their cognate precursor and leader peptides, none of the polypeptides contain an identifiable RRE.

Some lanthipeptides also contain the α,α-disubstituted amino acid labionin, that is installed by a second Michael-type conjugate addition of the enolate formed after cyclization with a free Dha (Figure S1).^30^ To date, almost all labionin linked lanthipeptides are produced by class III LanKC enzymes. Biosynthesis of this α,α-disubstituted amino acid by the LanKCs is particularly unusual as these enzymes lack the otherwise strictly conserved and catalytically requisite zinc ligands found across all other cyclase domains.^31^

Despite advances, there are significant knowledge gaps in the current understanding of the structure and mechanism of class III and IV lanthipeptide synthetases. To date, there have been no structure–function studies on any class III enzymes. The recent structure of the isolated kinase domain (encompassing amino acids 128–489 of the full length 866 residue polypeptide) of the class III CurKC reveals an expected kinase fold.^32^ However, details of the interactions between the domain and of interactions with nucleotide triphosphates are sparse. In addition, there is no experimental data on how the leader peptide is engaged in the absence of an RRE. Moreover, it is unclear if and how leader binding can mediate activation of the three (namely, kinase, lyase, and cyclase) active sites. There is no information on how class III enzymes can carry out cyclization reactions in the absence of a metal ion, which is required for catalysis in the other class I–II and IV metal-dependent enzymes. Lastly, it is unclear how the class III enzymes mediate the formation of labionin rings. Hence, information about the mechanism of substrate processing by class III enzymes remains limited.

Here, we carried out *in vitro* reconstitution of a class III lanthipeptide biosynthetic pathway from *Bacillus thuringiensis* NRRL B-23139, including the identification of a two-component protease system for leader peptide removal. We present the first high resolution crystal structures of a class III LanKC (2.5 Å resolution), as well as complexes with ATP and leader peptide, provided in trans (2.5 Å resolution) or as a single-chain fusion (2.15 Å resolution). We show through structure-guided biochemical studies that binding of the leader results in conformational changes that organize interdomain interactions to facilitate catalysis. Additional structure–function analysis suggests how the cyclase domain can facilitate thioether ring formation in the absence of a metal cofactor and also informs on residues that may help facilitate formation of labionin rings. These studies inform on how class III lanthipeptide synthetases make use of interactions between three different active sites for lanthionine or labionin ring formation.

## Results and Discussion

### Reconstitution of Viable Class III Lanthipeptide Biosynthetic Pathways

Numerous candidate class III lanthipeptide biosynthetic enzymes were systematically screened for biochemical activity and for solubility either in isolation and in complex with cognate leader peptides or full-length precursor peptides. Promising candidates that behaved well were the enzymes from a class III lanthipeptide biosynthetic pathway from the firmicute *Bacillus thuringiensis* NRRL B-23139. This biosynthetic gene cluster encodes for seven different precursor peptides (ThurA_1_–A_7_), which are nearly sequence identical, a *S*-adenosylmethionine (SAM)-dependent methyltransferase (hereafter ThurMet; GenBank accession WP_172554314), and a class III LanKC (hereafter ThurKC, WP_172554310) (Figure 1C). One product of this gene cluster (andalusicin, derived from precursor peptide ThurA_1_) has been previously characterized as a methylated class III lanthipeptide with narrow spectrum antibacterial bioactive against *B. cereus* ATCC4342.^33^ The isolated natural product contains 11 dehydroamino acids, three of which are part of a labionin ring, along with two methylations on the α-amine (Figure 1C).

Heterologous coexpression in *E. coli* of ThurKC along with the His_6_-tagged ThurA_1_ precursor peptide (WP_172554311) facilitated milligram level production of the complex. To test whether ThurKC was functional, the dehydratase and cyclase activities of the enzyme were tested. For *in vivo* characterization, the tagged modified precursor was first affinity purified and the MBP-His_6_ tag was removed using tobacco etch virus (TEV) protease. Reconstitution efforts focused on ThurA_1_ and ThurA_7_ (WP_172554313) as these are divergent in their respective core sequences (Figure S2). The biosynthetic gene cluster lacked the requisite protease for leader peptide removal, but prior bioinformatics analysis of class III lanthipeptide proteases characterized two M16B family zinc–metallopeptidases (GenBank entries WP_061520580.1 and WP_061520579.1) that are found in other class III clusters. Genome-wide interrogation of *B. thuringiensis* NRRL B-23139 identified four candidate M16B peptidases ThurP1 (WP_048545971.1), ThurP2 (WP_172554144.1), ThurP3 (WP_172554699.1), and ThurP4 (WP_172554700.1) outside of the genomic cluster. Coexpression of His_6_-tagged ThurP3 with untagged ThurP4 in *E. coli* yielded a stable complex that remained associated through multiple chromatographic steps. The recombinant two-component protease (ThurP3/P4) is used to remove the leader peptide in all subsequent experiments detailed below the full-length peptide. Incubation of modified ThurA_1_ or ThurA_7_ peptides ThurP3/P4 resulted in facile removal of the leader sequence (Figure 1D). The leader-free modified peptides were precipitated and further purified by using reverse phase HPLC.

Matrix-assisted laser desorption/ionization coupled to time-of-flight mass spectrometric (MALDI-TOF MS) analysis of the purified peptide was consistent with the removal of 11 water molecules (dehydrations) from the parent sequence (Figure 1D). As formation of lanthionine/labionin rings are mass neutral, the modified peptide was incubated with β-mercaptoethanol (βME), which is reactive toward accessible electrophilic dehydroamino acids. MALDI-TOF analysis demonstrates that βME readily formed adducts with nine dehydroamino acid residues, suggesting that the two remaining dehydroamino acids may have formed labionin rings (Figure 1D). Tandem mass spectral analysis of the modified peptide shows that residues following Ala16 in the precursor peptide cannot be fragmented, and this is consistent with the ring structure observed in characterization of the isolated natural product (Figures S3 and S4).

Incubation of modified ThurA_1_ or ThurA_7_ after the removal of their leader sequences with recombinant ThurMet methyltransferase and SAM yielded products with mass changes corresponding to the addition of two methyl groups (Figure S5). These studies demonstrate the expected activities of the enzymes necessary for production of the class III lanthipeptide andalusicin and its congener and identifies the two M16B metallopeptidases as the cognate leader proteases.

### Leader Peptide Fusion Yields a Constitutively Active Class III Lanthipeptide Synthetase

To date, there are no crystal structures of any class III lanthipeptide synthetases. We pursued structural studies of ThurKC either by itself, in complex with the full-length precursor peptide ThurA_1_, or with a peptide containing the leader sequence. These efforts yielded small and thin crystals but with variable diffraction quality. Despite inconsistent diffraction, a small number of crystals were suitable for data collection and allowed for data sets with resolutions of up to 2.5 Å to be collected. Crystallographic phases could not be obtained despite extensive screening of various heavy atom derivatives, mainly due to issues with variations in the diffraction quality of the crystals. Reasoning that heterogeneity in the stoichiometry of ThurKC with bound precursor peptide may contribute to the poor crystal quality, we generate a single-chain fusion of the ThurA_1_ leader peptide attached to the N-terminus of ThurKC with poly(-Gly-Ser-) linkers of various lengths. The most suitable fusions consisted of either seven or ten residues separating the leader peptide and ThurKC, hereafter termed LP-(GS)_X_-ThurKC, where X represents the number of residues in the linker.

Each of these fusion constructs was tested to determine if they could catalyze *in vitro* ATP-dependent dehydration of peptide substrates that lacked the leader sequence. Attempts to purify the full-length unmodified core peptide as a substrate were not successful due to degradation, with the major degradation product consisting of the first 14 modified residues of the ThurA_1_ core sequence. Hence, we carried out activity studies using a precursor peptide truncated at Val14 (hereafter, ΔC8 ThurA_1_). Incubation of wild-type ThurKC with ΔC8 ThurA_1_ produced a peptide product that had undergone the expected eight dehydrations, demonstrating that the truncated peptide is a substrate for dehydroamino acid formation (Figure S6). The same precursor peptide was modified when coexpressed with wild-type ThurKC, confirming that *in vivo* and *in vitro* experiments produced the same product (Figure S7).

**Figure 2:**
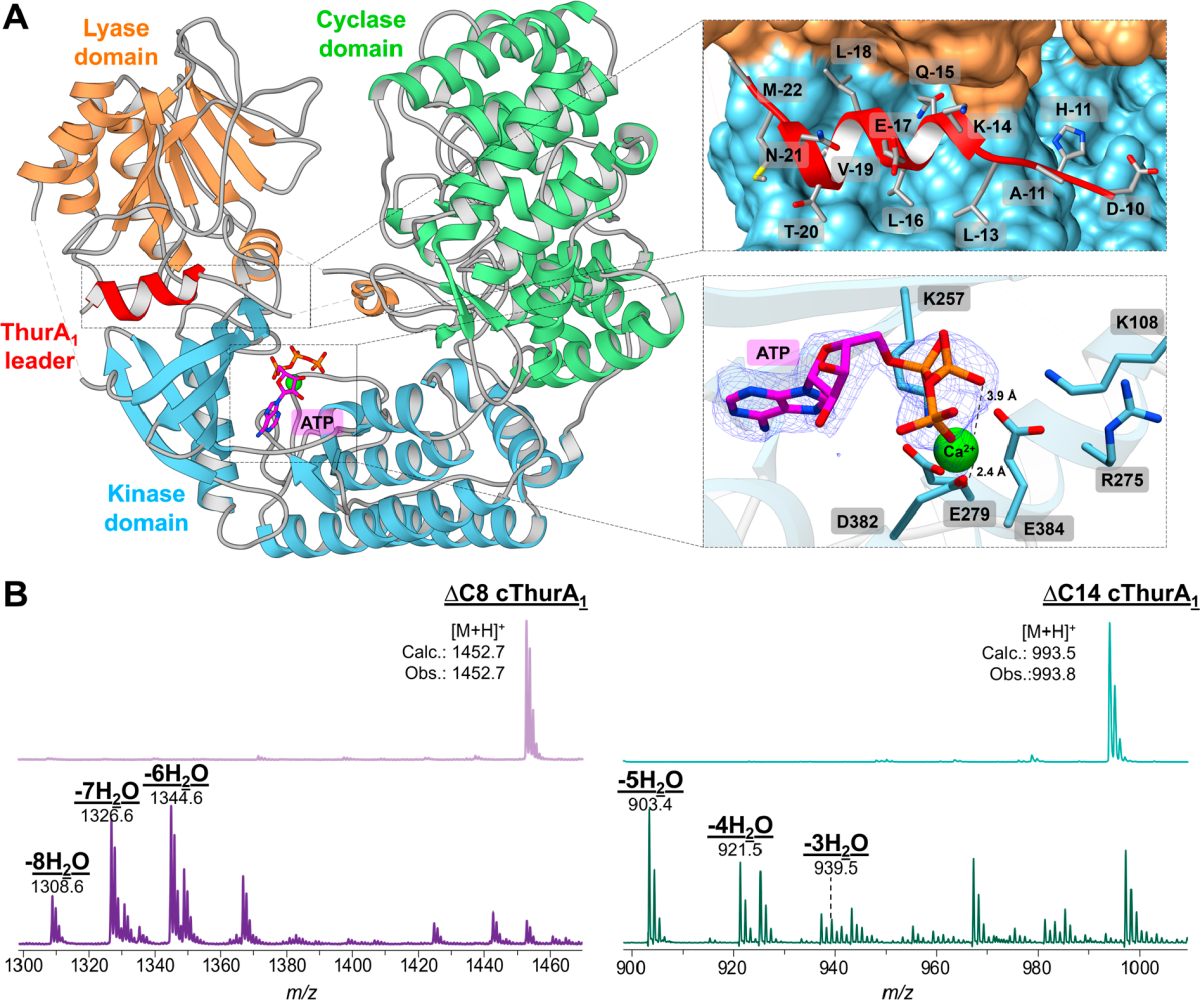
The LP-(GS)_7_-ThurKC fused enzyme construct is bound to the leader and active on the free core. (A) Structure of LP-(GS)_7_-ThurKC bound to ATP and MgCl_2_. Top inset shows the binding site of ThurA_1_ leader binding pocket in surface representation of ThurKC. Bottom inset shows a simulated annealing omit map (contoured at 2.7σ above background) calculated with Fourier coefficients (Fobs – Fcalc) with phases from the final LP-(GS)_7_-ThurKC models minus the coordinates of ATP omitted prior to refinement. (B) *In vitro* modification of ThurA_1_ truncated peptide core by LP-(GS)_7_-ThurKC. Synthetic peptides corresponding to residues 1–9 (right) and 1–14 (left) of ThurA_1_ were incubated in the presence of LP-GS_7_-ThurKC, 5 mM ATP, and 5 mM MgCl_2_.

Next, we tested *in vitro* dehydration activities of various single-chain leader fused ThurKCs using only the core peptides consisting of either the first 14 residues or the first 9 residues. Incubation of either LP-(GS)_7_-ThurKC with the 14-residue core peptide in the presence of ATP and MgCl_2_ produced a product with the expected 8 dehydrations, although the major products contained 6 or 7 dehydrations. Incubation with the 9-residue core peptide also produced dehydration products albeit much less efficiently (Figure 2B). As neither of the short peptides contained the terminal Cys(23), they would not be expected to contain labionin or lanthionine rings. Attempts to coexpress the ThurA_1_ core with the single-chain fusions resulted in incomplete dehydration, making it difficult to separate intermediates and characterize ring formation (Figure S8). Nonetheless, these experiments show fusion of the leader sequence to the ThurKC lanthipeptide synthetase yielded a constitutively active enzyme that could act on peptide substrates without a leader *in vitro* and *in vivo*, as has been demonstrated for a leader peptide-catalytic domain fusion of class II LctM^34^ and the unrelated YcaO heterocyclases involved in cyanobactin biosynthesis.^28^

### Crystal Structure of a Class III LanKC Synthetase

The single-chain LP-(GS)_7_-ThurKC fusion yielded easily reproducible crystals that reproducibly diffracted to resolutions beyond 2.5 Å. Crystallographic phases were determined by single-wavelength anomalous diffraction methods using data collected on the selenomethionine-substituted enzyme. A preliminary model consisting of ∼70% of the scattering atoms was used to phase and build using a 2.15 Å resolution native data set collected on crystals of LP-(GS)_7_-ThurKC bound to ATP and Ca^2+^ (as a surrogate for Mg^2+^) (Figure 2A). There are two copies in the crystallographic asymmetric unit and continuous density can be observed for both polypeptides throughout their entire lengths except for segments Asp(−10) of leader peptide to Glu2 of ThurKC, residues Arg24–Asn28, and Ser214–Glu222, which are all presumably disordered.

The overall structure of LP-(GS)_7_-ThurKC resembles a cupped hand with the lyase and kinase domains forming the fingers and the cyclase domain forming the palm and thumb (Figure 2A). The lyase domain is composed of residues Ile29 through Asn213, the kinase domain consists of residues Ser233 through Pro478, and the cyclase domain consists of residues Asn491 through Val862. While the overall architecture of the canonical folds for each of these domains can be easily identified, there are numerous insertions in each of the three domains that likely facilitate communication between the three domains with the peptide substrate. There are two additional regions of consequence in the structure that are discussed at length. First, clear and continuous electron density can be observed for the leader peptide corresponding to residues Met(−22) to Asp(−10), and this accounts for all but the C-terminal ten residues of the leader peptide (Figure 2A). Lastly, residues Asn4 through Ile21 form a short α-helix (hereafter, the N-helix) followed by a turn and interact with all three domains, suggesting that it allows for functional interplay between the domains.

### A Lyase Domain Adapted from Bacterial Virulence Effectors

The lyase domain of ThurKC adopts a compact α/β fold that is reminiscent of that of the HopA1 family of type III secretion system virulence effectors (Pfam PF17914) that catalyze the β-elimination of phosphate from phosphothreonine (pThr).^35^ A DALI search^36^ of the isolated lyase domain against the Protein Data Bank (PDB)^37^ reveals the closest structural homologues to be the type III effector SpvC from *Salmonella enterica* (PDB Code 2Q8Y; *Z*-score of 11.4 and 144 Cα residues aligned with an RMSD of 2.6 Å),^38^ OspF from *Shigella flexneri* (PDB Code 3I0U; *Z*-score of 11.3 and 137 Cα residues aligned with an RMSD of 2.6 Å), the HopA1 effector from *Pseudomonas syringae* (PDB Code 4RSW; *Z*-score of 9.5 and 139 Cα residues aligned with an RMSD of 3.5 Å),^39^ among others.

A superposition of the cocrystal structure of SpvC bound to a phosphopeptide derived from human ERK2 (PDB Code 2Z8P)^40^ shows near conservation in ThurKC of residues that are important for pThr recognition by SpvC (Figure S9). Four invariant residues in SpvC are involved in interactions with the phosphate group of the pThr substrate, and these include Lys104, Arg148, Arg213, and Arg220. In the structure of ThurKC, Lys65 and Arg163 are aligned well with the SpvC counterparts. Each of these 2 residues are vital for Dhb/Dha modification, given that no turnover is observed in ThurKC variants K65A and R163A (Figure S10). However, residues equivalent to Arg148 and Arg220 of SpvC are displaced due to insertions within the secondary structure in the ThurKC lyase domain. Each of these residues undergo large scale movement to accommodate substrate binding by SpvC. In the case of ThurKC, the positions of the corresponding residues are fixed by the secondary structural insertions and fix the positions of Lys117 (corresponding to Arg148) and Arg191 (corresponding to Arg220) toward the active site even in the absence of bound phosphopeptide. Although the K117A variant produces 11 dehydrations on ThurA_1_, it stalls in processing and shows mostly products with 7–10 dehydrations. *In vivo* processing of ThurA_1_ with the R191A variant results in phosphorylated intermediates (Figure S10). Lastly, His106, Lys136, and Asp201 of SpvC that are presumed to be involved in acid/base catalysis are conserved in ThurKC as His67, Lys94, and Asp152. The H67A variant stalls and shows many dehydrated and phosphorylated intermediates, while the K94A variant only showed completely unmodified peptide mass peak. The D152A variant can only form a maximum of 5 Dha/Dhbs, while most of the ThurA_1_ peptide remains unmodified (Figure S10). Residues Lys65, His67, Lys94, Lys108, Lys117, Asp152, Arg163, and Arg191 are closely conserved in Class III lanthionine synthases that have been biochemically characterized to produce labionin containing lanthipeptides, supporting their proposed catalytic roles (Figure S11).

### Regulation of an Activated Ser/Thr Kinase

The kinase domain of ThurKC adopts the canonical bilobular kinase fold with a deep intervening cleft as first described in the structure of cyclic AMP-dependent protein kinase.^41^ The N-lobe (Ser233 to Glu310) is formed by five β strands with an α helix called the C-helix (αC), and the C-lobe is made up of six α helices (Gly314 to Pro478). A DALI search against the PDB identifies several Ser/Thr protein kinases as structural homologues including 3-phosphoinositide-dependent protein kinase-1 (PDK1) (PDB Code 1H1W; *Z*-score of 20.1 and 215 Cα residues aligned with an RMSD of 2.4 Å),^42^ dual-specificity tyrosine phosphorylation-regulated kinase (DYRK1A) (PDB Code 7A4W; *Z*-score of 19.9 and 222 Cα residues aligned with an RMSD of 2.4 Å),^43^ death associated protein kinase (DAPK) (PDB Code 1JKS; *Z*-score of 19.8 and 212 Cα residues aligned with an RMSD of 2.6 Å),^44^ among several others. As in structures of other protein kinases, the ATP binding site in ThurKC is located at the juncture between the N- and C-lobes.^5^

All protein kinases contain an activation segment of 20–35 residues that begins with a highly conserved Asp-Phe-Gly (DFG) motif and terminates with a less conserved Ala-Phe-Glu (APE) motif.^45−47^ The equivalent region in ThurKC corresponds to a long loop that stretches from Asp382 through Lys408. In active kinases, the DFG motif serves as the N-terminal anchoring point for the activation loop and the Phe residue packs between two hydrophobic residues in helix (αC).^45^ A structural superposition with the proto-oncogenic Ser/Thr protein (PIM)-1 kinase (PDB Code 1YXT) shows that Asp382, Phe383, and Glu384 form the DF(G) motif in ThurKC (Figure S12).^48^ In the active conformation, Asp from the DFG motif is in the proximity and orientation to bind a magnesium ion that interacts with the oxygen atom of the β phosphate of ATP to facilitate phosphate transfer (Figure 2A). The DFE motif is conserved in Class III lanthionine synthases that have been biochemically characterized to produce labionin containing lanthipeptides (Figure S13). While this motif has also been identified in the structure of the isolated kinase domain of CurKC, the predicted model for the binding of nucleotide triphosphate and metal are not consistent with our experimentally determined structure.^32^

**Figure 3:**
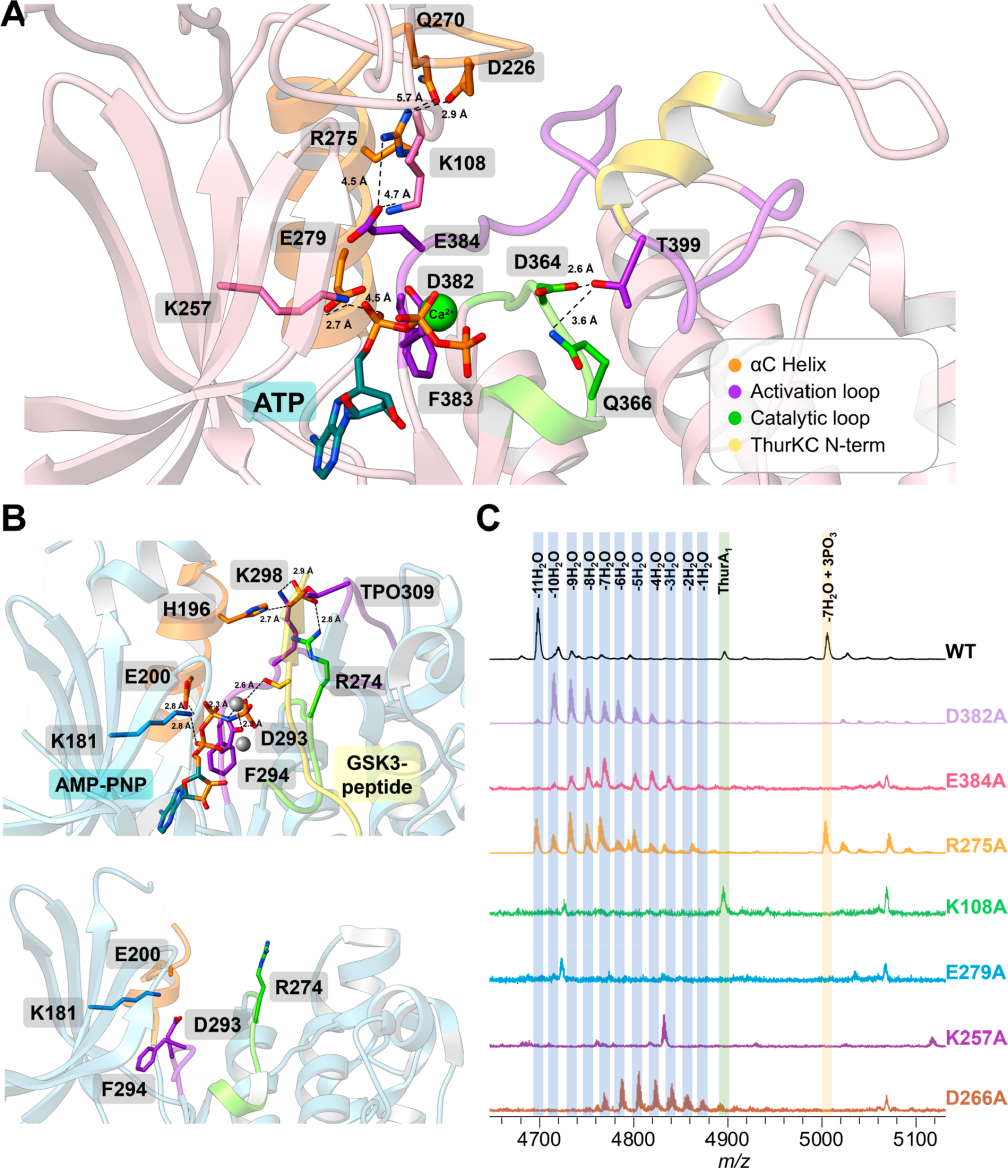
Structures of ThuKC. (A) Crystal structure of the ThurKC kinase domain bound to the Ca^2+^ ion (dark gray) and ATP (turquoise). The catalytic loop is colored in light green, and the activation loop in purple with the DFE residues shown as sticks and with the E384 substitution closely interacting with R275 and K108 in the lyase domain. The helix corresponding to the αC helix is orange, and the residues in the loop preceding it, D266, Q270, and R275, set the insertion in place. The N-terminal lyase helix that aids in positioning the rest of the activation loop is yellow. (B) The structure of an active PKB (1O6L, top) displays the position of the activation (purple), catalytic (light green) loops, and αC helix (orange). The synthetic hybrid peptide substrate (termed GSK3-peptide) is shown in yellow, and the inert nucleotide analog AMP-PNP is shown in turquoise. The residues vital for holding the active conformation and interaction with the catalytic loop are shown as sticks. In the inactive PKB structure (1GZN), the activation loop (purple) and αC helix (orange) are disordered and do not interact with the catalytic loop. (C) Heterologous coexpression of ThurA_1_ peptide with ThurKC lyase domain mutants in *E. coli* and analysis by MALDI-TOF MS.

The replacement of the canonical Gly with Glu384 in the DF(G/E) motif of ThurKC results in the positioning of the activation segment and Arg275 in the kinase domain in the vicinity of Lys108 in the lyase domain, albeit at a ∼4.5 Å distance that is too far for a direct interaction (Figure 3A). Notably, Lys108 is found at the start of a loop that contains the catalytically important Lys117 in the lyase domain, and the loop that includes Lys108 and Lys117 is absent in the structures of other pThr lyases such as SpvC and OspF. This loop insertion in ThurKC may allow for interactions between the lyase domain and the DF(G/E) motif in the kinase domain to possibly orient both active sites. The K108A variant does not install any Dha or Dhbs, hinting that Lys108 may play some role in modulating dehydratase activity (Figure 3A). The E384A variant shows major peptide degradation, and only 7 out of the 11 possible Dha/Dhb residues are observed. The R275A variant can form all 11 Dha/Dhb, but with several intermediate products observed, ranging from 4 to 11 Dhb/Dha residues. The compromised activity of these three variants suggests that interactions between Glu384 of the DF(G/E) motif and Lys108 and Arg275 may help to stabilize the activation segment in ThurKC.

A second feature of protein kinases in the activated state is an inward disposition of the helix αC, which positions a conserved Glu in a hydrogen bond with a conserved Lys from strand β3.^46^ This interaction, along with the hydrophobic packing of the DF(G/E) motif, is characteristic as it positions the Lys for interactions with the ATP β phosphate. In the structure of ThurKC, Glu279 from αC is positioned within hydrogen-bonding distance to Lys257. Mutating Glu279 and Lys257 to Ala in ThurKC produced degraded peptide fragments upon coexpression with ThurA_1_, consistent with the importance of these interactions across all activated kinases (Figure 3C). In all protein kinases, the C-terminal anchoring point of the activation loops contains residues that form the binding interface of the respective peptide substrate. This anchor starts at the P+1 loop of the kinase, which is a critical region of contact with substrate. Although the position of the C-terminal anchor is nearly identical, sequence differences define the distinct chemo specificities between the Ser/Thr and Tyr kinases. As in the structures of activated Ser/Thr kinases, a conserved Thr399 in ThurKC hydrogen bonds with residues in the catalytic loop (Asp364 and Asn366) that position this residue in the substrate for phosphoryl transfer. In Tyr kinases, a proline residue replaces this conserved Thr.

In kinases that are activated by phosphorylation, termed RD kinases (such as PKB/Akt), phosphorylation of a Ser or Thr in the activation segment configures the active form of the enzyme (Figure 3B).^49^ In the structure of RD kinases, Ser/Thr phosphorylation enables interactions between the phosphate group and residues in helix αC, in the catalytic loop, and in the activation segment. For example, in the structure of activated PKB/Akt, His196 from helix αC, Arg274 from the catalytic loop, and Lys298 from the activation segment all contact pThr309, thereby orienting spatially distinct regions of the enzyme to shape the activated conformation.^49^ In contrast, ThurKC contains an ∼8 residue insertion spanning Pro261 through Asp271 that precedes helix αC and is in contact with the activation segment (Figure 3A). The configuration of this insertion is set by hydrogen bonding interactions between Asp266 and Gln270 and Arg275. The opposite side of the activation helix is fixed through interactions with an N-helix spanning residues Met5 through Leu11 that precedes the lyase domain. As a result of these interactions both within the kinase domain and with residues in the N-helix, the orientation of the activation segment is fixed and is consistent with that observed in activated forms of other protein kinases such as PKB/Akt and the constitutively activated PIM-1.^48,50,51^ A ThurKC variant with deletion of the first 33 residues of the N-terminal helix (ΔN33 ThurKC) is unable to modify the ΔC8 ThurA_1_ precursor peptide *in vitro*. This variant was also unable to produce fully modified ThurA_1_*in vivo*, stalling in products ranging from 8 to 10 dehydrations, and unmodified and phosphorylated intermediates (Figure S14). These data confirm the importance of the N-helix of ThurKC in orienting the activation segment in an activated conformation.

### A Metal Independent Cyclase Domain for (Me)Lan Formation

The structure of the ThurKC cyclization domain exhibits the α,α-toroidal fold that has previously been observed in structure of isolated class I LanC cyclases and in the cyclization domain of fused class II LanMs.^14^ A DALI search against the PDB identifies the closest structural homologues as NisC (PDB Code 2G0D; *Z*-score of 28.0 and 321 Cα residues aligned with an RMSD of 3.6 Å),^17^ the cyclase domain of CylM (PDB Code 5DZT; *Z*-score of 22.4 and 274 Cα residues aligned with an RMSD of 3.0 Å),^19^ and the eukaryotic LanC-like proteins LanCL1 (PDB Code 3E6U; *Z*-score of 28.1 and 320 Cα residues aligned with an RMSD of 3.1 Å)^52^ and LanCL2 (PDB Code 6WQL; *Z*-score of 29.6 and 318 Cα residues aligned with an RMSD of 2.9 Å).^53^ Prior sequence-based prediction demonstrated that the cyclase domains from LanKCs lacked the canonical zinc ligands, and experimental data are consistent with a metal independent strategy for class III lanthipeptide cyclization.

**Figure 4:**
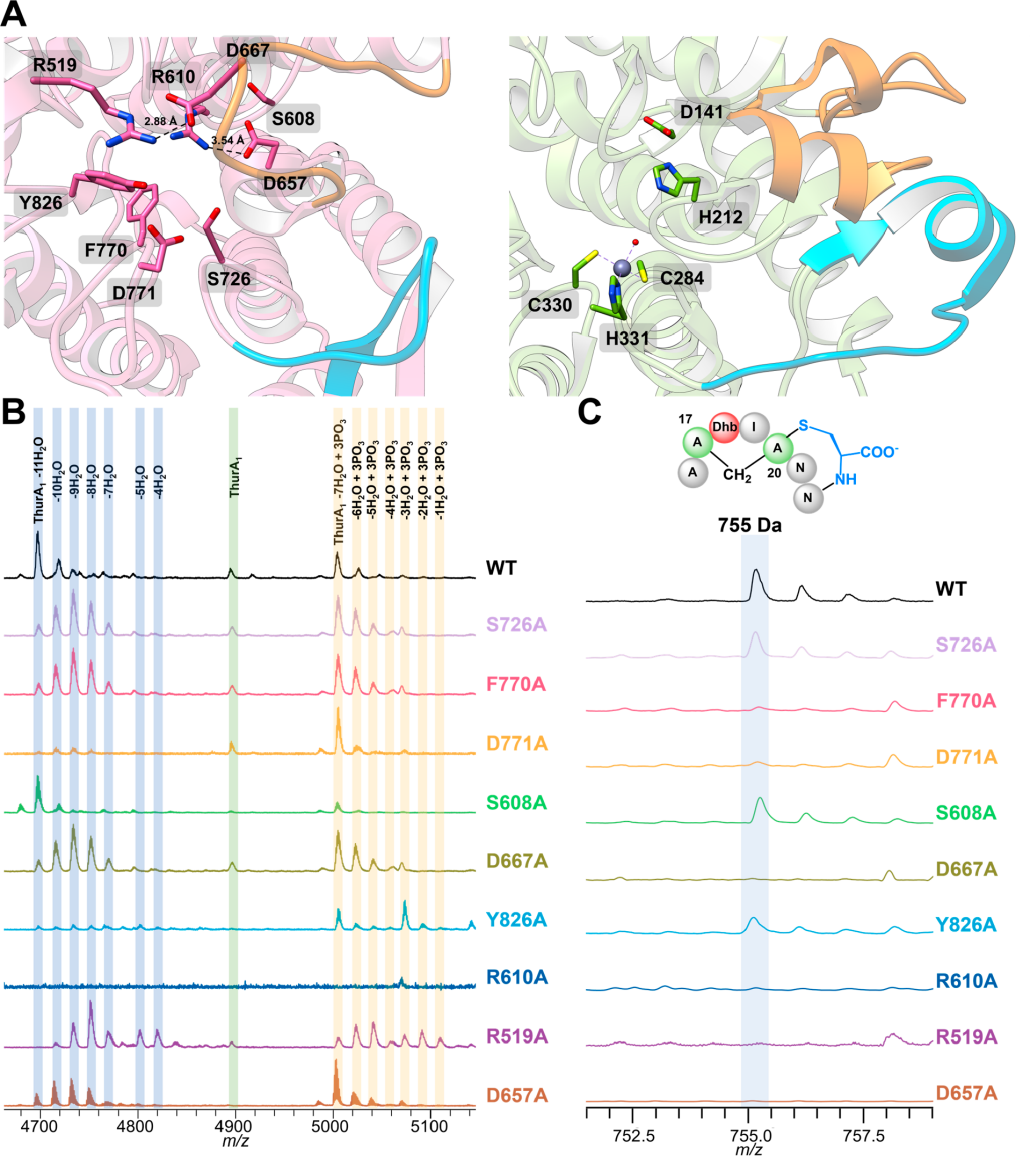
Class III cyclase domain structure. (A) The ThurKC cyclase domain (left) is shown next to class I cyclase NisC (right). The zinc binding residues in NisC are not present in the ThurKC cyclase. SH2-like insertions are shown in orange and blue in NisC, and the equivalent residues in ThurKC are shown for comparison. (B) Heterologous coexpression of ThurA_1_ peptide with ThurKC cyclase domain mutants in *E. coli* and analysis by MALDI-TOF MS. Surprisingly, most mutations in the cyclase domain impact dehydroamino acid production. (C) Digestion of these ThurA_1_ products with proteinase K is used to determine labionin formation.

A superposition of the structures ThurKC with the zinc-dependent class I nisin cyclase NisC shows that the Cys284, Cys330, and His331 zinc ligands have been replaced with Ser726, Phe770, and Asp771, respectively, and no evidence for electron density corresponding to a metal ion is observed in any of the ThurKC structures (Figure 4A). In metal-dependent LanC type cyclases, the catalytically requisite zinc functions as a Lewis acid to lower the p*K*_a_ of Cys thiol conjugate addition on the electrophilic dehydroamino acid.^54^ The metal may also orient the resulting thiolate for a nucleophilic attack on the electrophile. It is plausible to consider that Asp771 of ThurKC may function as a general base in Cys deprotonation and Phe770 may help orient the thiolate for productive attack. The F770A variant of ThurKC produced a minor amount of the fully dehydrated product when coexpressed with ThurA_1_ but mostly stalled at 7–10 dehydrations or 7 dehydrations and 3 phosphorylations (Figure 4B). To determine whether any of the intermediate products of the mutant ThurKC contained a labionin or lanthionine ring, the ThurA_1_ peptide, modified by WT ThurKC and leader sequence excised using the ThurP3/P4 leader protease, was digested using proteinase K. For the fully modified ThurA_1_ peptide, the labionin region and the amino terminal alanine are protected from further degradation with a mass of 755 Da (Figure 4C). When the ThurA_1_ intermediate peptide mixture produced by ThurKC F770A was subjected to proteinase K treatment, the 755 Da peak was not present, hinting at the importance of this residue in lanthionine or labionin ring formation. Alanine replacements at the other two residues in place of the canonical zinc ligands also yielded products with an incomplete number of dehydrations. The S726A variant produced minor quantities of product with 11 dehydrations and mostly produced an intermediate with 9 dehydrations and smaller amounts of other stalled dehydration products. The proteinase K degradation of this product does reveal the 755 Da peak, indicating that labionin cyclization had occurred. Lastly, the D771A variant mostly produces an intermediate of ThurA_1_ with 7 dehydrations and 3 phosphorylations. When treated with proteinase K, the intermediate mixture produced by D771A ThurKC did not show a peak corresponding to a protected labionin ring (Figure 4C).

Canonical zinc-dependent cyclases contain two highly conserved residues (Asp141 and His212, NisC numbering) that are implicated in acid–base catalysis. In other LanC enzymes, highly conserved and functionally essential residues Asp141 and His212 are proposed to protonate the enolate that is formed after conjugate addition.^14^ In the structure of ThurKC, these residues are replaced with Ser608 and Asp667, respectively. The S608A variant produced a peptide with all 11 dehydrations as the primary product, and proteinase K treatment reveals that this fully dehydrated peptide also contains a labionin (Figure 4B,C). The D667A variant can produce fully dehydrated ThurA_1_ but mostly produces stalled intermediates with 9 dehydrations. Notably, this variant fails to produce a mass consistent with a labionin peak upon proteolysis.

During the formation of a labionin ring, the initially formed enolate attacks a different dehydroamino acid to form a second enolate, which is then protonated by a general base (Figure S1). In the structure of ThurKC, Arg610 is in proximity to the position of His212 in the structure of NisC, and the orientation of this side chain is set by a salt bridge with Asp667. Mutations of either Arg610 or Asp667 impede complete dehydration: the R610A variant results in complete disappearance of the ThurA_1_ in all forms, likely due to degradation, while the D667A variant mostly stalls at 8, 9, and 10 dehydrations and 7 dehydrations and 3 phosphorylations. Further analysis with proteinase K shows that neither the intermediate nor degraded peptide mixture products of R610A or D667A ThurKC produce a labionin ring (Figure 4C). Although these variants should have no effect on peptide turnover, these amino acids might be essential for contacts between the cyclase domain and the peptide substrate.

To provide a suitable proxy for how a substrate peptide may interact with the cyclase domain, we generated an Alphafold model of ThurKC bound to full-length ThurA_1_ peptide and compared this model to our recently determined cocrystal structure of a eukaryotic LanCL1 cyclase bound to glutathionine and a peptide derived from Erk.^54^ The comparison identified that a plausible basic residue that may protonate the second enolate that is formed after the carbon–carbon cross-link is formed during labionin formation is Arg519, which is positioned ∼6 Å away from Asp770 and Asp771. This residue is stabilized by a salt bridge with Asp657 (Figure S15). Alanine mutants of either of these two Arg residues also show stalled processing with only D657A able to produce ThurA_1_ with 11 dehydrations in a small amount. Neither of the ThurA_1_ intermediate mixtures of R519A nor D657A ThurKC show the labionin peak following proteinase K treatment. These two pairs of residues also appear to not be conserved through sequence alignments to labionin producing LanKCs, but superimpositions of their Alphafold models with the ThurKC structure show that an Arg or His residue is always present in the loop region of R610; in 75% of the models, there is an Asp or Glu residue in that same loop region (Figures S16–S18). However, R519 appears to be less conserved in positioning with only 37.5% of models having it in the same region, and the loop containing D657 is not modeled close to the D519 loop in most of the predicted models, although being present in the loop in 88% of the models, indicating potential flexibility in the predicted models.

### Binding Site of the Leader Peptide

Leader peptide residues Asp(−10) to Met(−22) are bound perpendicular to the antiparallel β-strands that form the N-lobe of the kinase domain (Figure 2A). A comparison with the structure of wild-type ThurKC bound to the leader peptide in trans (2.45 Å resolution) confirms the binding mode and rules out artifactual binding due to the single-chain fusion. The leader region binds as a three turn α-helix, encompassing residues Lys(−14) through Met(−22), and the helical configuration is consistent with prior multidimensional NMR studies of the synthetic leader peptides of class III lipolanthine from *M. arborescens* (MicA from the microvionin pathway).^55^ The remainder of the leader sequence (Leu(−13) through Asp(−10)) exists in an extended form and is directed toward the kinase and lyase active sites. Some characterized RiPP biosynthetic enzymes contain a dedicated RiPP recognition element (RRE) that binds the leader as an extended β-strand.^29^ However, this is not a universal feature of all RiPP pathways, and several core biosynthetic enzymes lack an RRE-like domain. The class III LanKC is an example of the growing class of RiPP biosynthetic enzymes that can bind leader sequences without an RRE.^56−59^

The leader helix is amphipathic in nature with the hydrophobic residues directed toward contacts with the ThurKC and most of the polar residues facing toward the solvent. The leader binding groove in ThurKC is formed by several aromatic or aliphatic residues from both the kinase and the lyase domains that provide van der Waals contact with the peptide. Examples of such packing include both Met(−22) and Leu(−18) binding in a pocket created by Tyr148 from the lyase domain and Trp302 from the kinase domain; Val(−19) engaged in a pocket created by Trp302, Phe306 from the kinase domain, and Leu225, which is in the linker region between the kinase and lyase domains, and Leu(−18) is situated in a pocket created by Phe306 and Ile230 from the linker (Figure S19). The last of the helical residues from the leader that contacts the protein is Gln(−15), and this residue is engaged in hydrogen bonding interactions with Tyr243 and Glu258 from the kinase domain and Arg112 from the lyase domain.

The end of the leader helix is stabilized on one side by a loop from the lyase domain composed of residues Asp189 through Pro198 and is further directed by Phe194, which is contained in this loop. On the opposite side, the helix is stabilized by numerous residues from the N-lobe of the ThurKC kinase domain, equivalent to the LRD domain identified in the CurKC as being essential for binding through MST binding assays.^32^ Notably, an aromatic residue located at the very start of the kinase domain (Phe236) disrupts the helical configuration, resulting in the remainder of the leader being extended as a coil that may enable shuttling between the different actives sites of ThurKC. Ala-scanning analysis of the ThurA_1_ precursor peptide shows that the M(−22)A variant results in triphosphorylated intermediates with 5 or 4 dehydrations. Mutations in the leader at Val(−19), Leu(−18), or Leu(−16) resulted in stalled processivity, with mostly 8–10 dehydroamino acids maximum (Figure S20). Mutant leader ThurA_1_ Q(−15)A shows stalling in Dha formation but can make 11 dehydroamino acids, and L(−13)A had no effect on dehydration.

Several structural elements ensure that the core peptide is directed toward the active site(s) of ThurKC rather than toward solvent (Figure S21). First, a large loop encompassing residues Thr714 through Leu722 from the cyclase domain, which is equivalent to the SH2-like extension found in NisC, extends toward the tail of the leader peptide and forms a buttress the core toward the kinase active site. Second, the N-helix forms a support at the opposite face of the leader entry pathway where it likely directs the core toward the lyase and cyclase active sites. Lastly, a loop that extends between Leu653 through Asp662 caps the trajectory of the peptide toward the cyclase domain. The three domains function in concert to direct the core peptide through the three active site to ensure the maturation of the modified peptide.

### Leader Binding Configures the Kinase Domain in the Active Form

**Figure 5:**
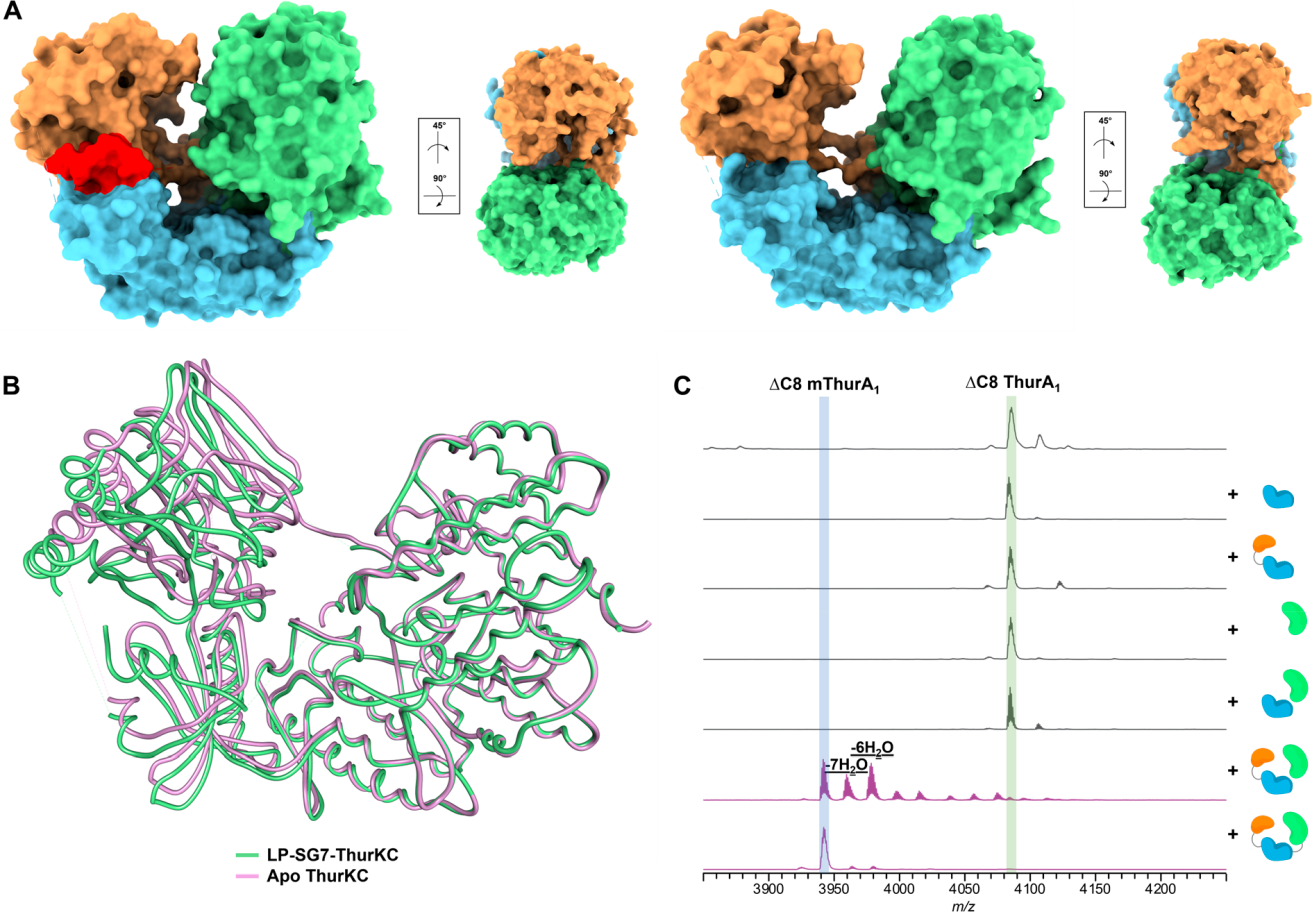
ThurKC activity is dependent on interdomain interactions. (A) Comparison of the surface structure of LP-(GS)_7_-ThurKC (left) with apo surface ThurKC (right) reveals shifts upon binding of the leader that orient the multiple active sites in a productive conformation. (B) Superposition of the structures of the cyclase domains of LP-(GS)_7_-ThurKC (green) with apo surface ThurKC (purple) shows that leader binding induces movements in the lyase and kinase domains. (C) In a split *in vitro* reaction with the truncated substrate Leader-ΔC8 ThurA_1_, the kinase domain (blue) is unable to phosphorylate the precursor peptide, even in the presence of the cyclase domain (green). Despite the ThurA_1_ leader binding in the groove between the lyase and kinase domain, the single polypeptide of these two domains (orange and blue) is not sufficient for dehydroamino acid formation. Dehydroamino acids are formed only when the lyase kinase domains and cyclase domains are present, even without a covalent linkage.

To compare the consequences of leader peptide binding on the structure of ThurKC, we carried out numerous attempts to produce the enzyme in the absence of the leader peptide, with no success. Expression of ThurKC in the absence of the leader peptide resulted in an impure protein that was prone to aggregation. Eventually, we were able to grow crystals of ThurKC that were purified with the leader but with most of the peptide removed during purification. The resultant 2.52 Å resolution structure does not show any density for the leader peptide at the binding site but may contain trace amounts that remained throughout the purification process. The structure of this “leaderless” ThurKC shows density for the activation segment and catalytic loops in conformations similar to that observed in the structure of LP-(GS)_7_-ThurKC. However, a comparison of the respective C-lobes of the two structures reveals that the N-lobe of “leaderless” ThurKC is shifted away from the C-lobe (Figure 5A,B). This movement opens the ATP and peptide-binding sites that are sandwiched between the two lobes. Although there are no changes in the orientation of secondary structural elements that define the activated conformation, the relative movement of the N- and C-lobes in the absence of the leader peptide shifts active site residues too far away to productively interact with ATP and presumably the peptide substrate. The displacement of the N- and C-lobes results in significant movements of both the lyase domain and the cyclase domain. Many of the residues that stabilize interactions between the three domains are displaced and incapable for interaction in the “leaderless” ThurKC structure.

### Functional Interplay between the Three Active Sites May Mediate Peptide Modifications

The structure of LP-(GS)_7_-ThurKC shows an interdomain interaction with ThurA_1_ leader peptide, and all three domains are predicted to contribute toward guiding the core peptide through each of the active sites for maturation. Additionally, Ala substitutions of ThurKC cyclase residues have been shown to impact its ability to catalyze the formation of dehydroamino acids in ThurA_1_ (Figure 4B). Seeking to understand the extent of this interdependence of activity, we cloned and expressed the individual kinase, lyase, and cyclase domains of ThurKC. The kinase domain (Ser223-Gln486) was soluble when coexpressed with ThurA_1_ peptide. We were unable to obtain a soluble lyase domain (ranging from Met1 to Ille211) but the lyase and kinase domains are soluble as a single polypeptide (Met1-Gln486). The cyclase domain (Thr487-Ile872) was also soluble when expressed with an N-terminal MBP fusion tag, which was removed with TEV protease. We could not produce suitable quantities of an unmodified full-length precursor despite multiple attempts and with different cloning and purification strategies. Hence, we set up *in vitro* reactions, with each of these subdomains using the leader-ΔC8 ThurA_1_ precursor peptide. The isolated kinase domain was unable to phosphorylate this peptide in the presence of ATP and MgCl_2_. Furthermore, the lyase-kinase fusion similarly was unable to modify this peptide, as neither phosphorylated intermediates nor dehydration final products could be observed. This contrasts with prior data showing activity for the isolated domains of other class III or class IV lanthipeptide synthetases.^32,60^ As the *in vitro* studies used a truncated substrate, we further sought to confirm the above observations with full-length ThurA_1_ precursor peptide using a coexpression strategy. As expected, wild-type ThurKC was able to effectively process the full-length precursor peptide, but coexpression with the excised lyase-kinase domain (Met1-Gln486) did not result in any modification to ThurA_1_ (Figure S22).

Notably, modifications on leader-ΔC8 ThurA_1_ could be observed when both the lyase-kinase and cyclase domains were present as separate polypeptides (Figure 5C). This in trans activity observed when the lyase-kinase and cyclase domains of ThurKC were mixed suggested that the polypeptides may form an active complex. We carried out analytical size exclusion chromatographic studies on wild-type ThurKC, the lyase-kinase domain, the cyclase domain, and a 1:1 mixture of lyase-kinase and cyclase domains. The lyase-kinase and cyclase domains did not form a stable complex with the expected molecular weight of full length ThurKC (Figure S23). We next added the leader-ΔC8 ThurA_1_ peptide to the lyase-kinase and cyclase mixture to determine if the precursor could induce the formation of a stable complex but did not see a shift in retention volumes. Hence, it is unclear as to why the activity is observed when the kinase-lyase and cyclase domains are added together. One plausible explanation is that transient interactions, which are not stable over the time course of chromatographic analyses, may occur between the domains. However, further biophysical experiments, such as FRET or STD-NMR, are needed to provide more direct evidence of complex formation.

## Conclusion

In this current work, we utilize a multipronged approach to understand the mechanics of lanthipeptide formation by a class III system. Full reconstitution of the biosynthetic pathway is enabled by the discovery of a new two-component zinc-dependent leader peptidase. We describe the crystal structure of ThurKC, the first of any for a full-length class III lanthipeptide synthase. The structural and corresponding biochemical analyses reveal numerous interactions between the lyase, kinase, and cyclase domains. Using a single-chain fusion of the leader peptide bound to the full-length ThurKC yielded a catalyst that can modify the core peptide as well as short fragments derived from the N-terminal sequence of the natural substrate.

The structures of ThurKC bound to the leader peptide, either as a single-chain fusion or in trans, identify the correct binding site for the leader in a class III system, which is distinct from previously predicted models. Biochemical studies identify a LELQL motif that is critical for interactions between the leader and ThurKC. Comparison of the ThurKC structure in the absence and presence of the leader peptide demonstrates that leader binding orients interactions between all three domains. This reorganization results in a constitutively activated kinase domain and reveals a new mode for activation for a kinase domain. Biochemical studies of site-directed variants show the critical role of long-range interactions in stabilizing the active conformation. Similar induced conformational changes upon leader binding have been observed in other orthogonal RiPP pathways, most notably in the cyanobactin cyclodehydratase LynD^28^ and the graspetide macrocyclase PsnB.^61^ Such reorganizations likely serve as a regulatory mechanism to prevent processing of noncognate peptides inside the producing organism.

An intriguing aspect of class III lanthipeptide synthetases is that they contain a cyclase domain that lacks any of the canonical metal-coordinating residues that engage the essential active site zinc in class I LanC cyclases or the cyclase domains from class II or IV lanthipeptide synthetases. In the ThurKC structure, the residues that occupy the same position as the zinc-binding ligands in the other systems are Ser726, Phe770, and Asp771. Mutational analysis of these residues shows that Ala replacement of either Phe770 or Asp771 yields variants that can carry out dehydration but not cyclization. Due to the variability in loop regions, simple sequence alignments do not clearly illustrate similarities in cyclase domains among class III LanKCs. We generated Alphafold models for the predicted structures of eight different LanKCs and superimposed these on our experimentally determined ThurKC structure. We identified an Asp in 87.5% and a Phe/Tyr/Met in 87.5% of the models, suggesting that these residues may be critical for orienting and deprotonating the substrate Cys for subsequent conjugate addition reactions to form a dehydroamino acid (Figure S18).

These studies also raise several new questions regarding the cyclization reaction, most notably: what dictates labionin formation in class III LanKCs and how does the substrate peptide toggle between the three active sites for productive catalysis? Attempts to obtain structures with bound core peptide by using inert nucleotide analogs, truncated precursor peptides, or inactive variants of either the kinase or lyase domain all failed to reveal any obvious density at the respective active sites. Nonetheless, the structural data presented here should provide a foundation for future biochemical or structural biological experiments to elaborate on these functionalities. Lastly, the single-chain LP-(GS)_7_-ThurKC fusion can catalyze dehydroamino acid formation on short peptide substrates and may be useful catalysts in biotechnology for generating analogs of other classes of RiPPs such as thiopeptides.

## Materials and Methods

Plasmids containing WT *thurKC* or site-directed mutants were constructed using the pET hexahis (His_6_) tobacco etch virus (TEV) protease ligation-independent cloning (LIC) vector (2S-T), a gift from Scott Gradia (Addgene plasmid #29711). Alanine mutants in the kinase, lyase, or cyclase domains of ThurKC were generated using site-directed, ligation-independent mutagenesis (SLIM).^62^ Detailed procedures for all mutagenesis studies are provided in the Supporting Information. All protein purification methods and synthetic details are provided in the Supporting Information.

## Supporting Information Available

Detailed methods for protein expression, purification, biochemical, and crystallographic studies, and additional data including tandem MS analyses, mass spectra, sequence alignment, MALDI-TOF MS analysis, and size exclusion chromatography (PDF)
