# Supplementary material for: Structure and Function of a Class III Metal-Independent Lanthipeptide Synthetase
Source: ACS Cent Sci. 2023 Oct 12;9(10):1944–56. doi: 10.1021/acscentsci.3c00484 (PMC10604976; doi:10.1021/acscentsci.3c00484)
Supplement: Supplementary file 1 — oc3c00484_si_001.pdf [file oc3c00484_si_001.pdf]

Structure and function of a class III metal-independent lanthipeptide synthetase

Andrea Hernandez Garcia[1] and Satish K. Nair[1,2,3*]

[1]Department of Biochemistry, [2]Center for Biophysics and Computational Biology, and [3]Carl R. Woese Institute for Genomic Biology

SUPPLEMENTARY FIGURE AND TABLE LEGENDS

Supplementary Methods S3
Table S1: Protein sequence accession numbers...S12
Table S2: Primers used in this study...S13
Table S3: Crystallographic statistics...S16
Figure S1: Biosynthetic scheme of labionin ring formation.......................................S17
Figure S2: Precursor peptides encoded in andalusicin BGC S18
Figure S3: Tandem MS analysis of reconstituted $ThurA_1$...S18
Figure S4: Tandem MS analysis of reconstituted $ThurA_7$...S19
Figure S5: Mass spectra of modified core $ThurA_1$ and $ThurA_7$ methylated by ThuMet............S20
Figure S6: Mass spectra of ΔC8 $ThurA_1$ modified *in vitro* by WT ThurKC.............................S21
Figure S7: Mass spectra of the leader peptide removal from dehydrated ΔC8 $ThurA_1$ S22
Figure S8: Mass spectra of *in vivo* modified $ThurA_1$ core by LP-GS_{10}-ThurKC......................S23
Figure S9: Comparison of the lyase domain of ThurKC to virulence factor SpvC....................S24
Figure S10: Mass spectra $ThurA_1$ co-expressed *in vivo* with ThurKC lyase variants................S25
Figure S11: Sequence alignment of lyase domains of class III LanKC reported to produce lanthipeptides containing labionin...S26
Figure S12: Comparison of the kinase domains of PIM-1 Kinase and ThurKC. S27
Figure S13: Sequence alignment of kinase domains of class III LanKC reported to produce lanthipeptides containing labionin...S28
Figure S14: Mass spectra $ThurA_1$ precursor peptides treated with ΔN33 ThurKC....................S29
Figure S15: Comparison of the cyclase domains of LanCL1 and ThurKC ……………….......S30
Figure S16: Alphafold active site predictions of LanKCs that have been biochemically characterized to produce labionin rings...S31
Figure S17: Sequence alignment of cyclase domains of class III LanKC reported to produce

lanthipeptides containing labionin.....S32
Figure S18: Alphafold predictions suggest conservation of crucial residues in the cyclase domain loop region of labionin producing LanKCs.....S33
Figure S19: Residues vital for ThurA$_1$ leader binding by ThurKC.....S34
Figure S20: Mass spectra ThurA$_1$ mutant leader products on co-expressions with ThurKC.....S35
Figure S21: Basis for orientation of the core peptide.....S36
Figure S22: MALDI-TOF MS analysis of ThurA$_1$ precursor peptide co-expressed with wild-type and truncated ThurKC.....S37
Figure S23: Analytical size exclusion chromatography of ThurKC and its subdomains.....S38
References.....S39

METHODS

Cloning, Heterologous Expression and Purification of ThurKC Proteins

Primers used for polymerase chain reaction (PCR) amplification are listed in Table S2. The gene encoding ThurKC (WP_172554310.1) was amplified from *Bacillus thuringiensis* sv. *andalousiensis* NRRL B23139 genomic DNA using primers designed based on the published sequence. The amplified PCR product was inserted into linearized pET His_6 tobacco etch virus (TEV) protease LIC cloning vector or pET His_6 MBP TEV LIC cloning vector (gifts from Scott Gradia, Addgene plasmid #29666 and #29656, respectively) using the Gibson Assembly Kit from New England Biosciences. Gibson assembled plasmids were maintained and propagated in *Escherichia coli* DH10β and the integrity of the gene was confirmed using dideoxy DNA sequencing. For protein production, the sequence-verified plasmids vectors were transformed into *E. coli* Rosetta (DE3) (Novagen) and plated on LB-Agar plates with ampicillin at 143 μM and chloramphenicol at 62 μM for the linearized pET His_6 TEV LIC ThurKC clone, and kanamycin at 52 μM and chloramphenicol at 62 μM for the pET His_6 MBP TEV LIC ThurKC clone. Single colonies were inoculated into LB media supplemented with the appropriate antibiotics grown at 37 °C to an OD_{600} of 0.6 before cooling in an ice bath ice for 15 minutes. Protein production was induced with the addition of 500 μM isopropyl β-D-1-thiogalactopyranoside (IPTG) and the cells were allowed to grow for additional 18 hours at 18 °C. Following growth, cells were harvested by centrifugation (3500 rpm, 20 min, 4 °C) and resuspended in suspension buffer (20 mM Tris–HCl, pH 8.0, 500 mM NaCl, and 10% glycerol). Attempts to grow ThurKC alone resulted in low levels of protein expression with numerous degradation fragments.

The gene for $ThurA_1$ was cloned into MCS2 of pRSFDuet-1 (Novagen plasmid #71341-3) and co-transformed with pET His_6 TEV LIC ThurKC into *E. coli* Rosetta (DE3) with the appropriate antibiotics (ampicillin at 143 μM, kanamycin at 52 μM, and chloramphenicol at 62 μM). Protein expression and production was carried out as described above, and cells were harvested by centrifugation and resuspended in suspension buffer. Cells were lysed by sonication with 30 second cycles at 40% amplitude with 1 min resting intervals, for a total of seven cycles. The crude lysate was centrifuged for 40 minutes at 14000 rpm and 4 °C to clear insoluble cell debris. The cleared lysate was passed through a 5 ml HisTrap nickel-nitrilotriacetic acid (Ni-NTA) affinity

column that was equilibrated with the suspension buffer. The column was washed with 50 mL of Wash Buffer (20 mM Tris–HCl pH 8.0, 500 mM NaCl, and 30 mM imidazole). ThurKC was eluted on an ÄKTA prime system (GE Healthcare Life Sciences) with a linear gradient over 40 mL, from 25 mM to 250 mM imidazole, at a flow rate of 2 mL min^{-1}. The purity of the eluted fractions was determined using SDS-PAGElectrophoresis. Pure fractions containing the protein were dialyzed overnight at 4 °C into dialysis buffer (300 mM NaCl, 20 mM Tris pH 8.0 and 10% glycerol) with the addition of TEV protease to remove the His_6 tag.

Subtractive purification using the His-Trap Ni-NTA column was used to remove the His_6 affinity tag, and an SDS-PAGE was used to confirm the purity of the tag-free protein samples. Proteins samples were concentrated in a 50 kDa Amicon (Millipore Sigma) and then further purified by size exclusion chromatography on a Superdex 200 column (GE Healthcare Lifesciences) equilibrated with gel filtration buffer (300 mM KCl and 20 mM 4-(2-hydroxyethyl)-1-piperazineethanesulfonic acid (HEPES) at pH 7.5). The protein was further concentrated with a 50 kDa Amicon and protein concentration was measured using a Nanodrop.

For production of the single chain fusion of the leader peptide with ThurKC, the $ThurA_1$ leader sequence was amplified using two rounds of PCR, one to add the SGSGSGS linker and the second to add an overhang designed for LIC cloning. The $ThurA_1$ Leader-SGSGSGS sequence was inserted into a linearized pET His_6 TEV LIC ThurKC vector between the affinity tag and the ThurKC coding sequence (hereafter LP-GS_x-ThurKC). The integrity of the plasmid was verified by DNA sequencing and then transformed into *E. coli* Rosetta (DE3) for protein expression as described above, with ampicillin at 143 μM and chloramphenicol at 62 μM. Different primers were used to extend the linker between the ThurA1 leader and ThurKC, with no discernible differences in protein yields. Selenomethionine incorporated LP-ThurKC was produced in minimal media using the method of metabolic suppression and purified in the same manner as described above for the pET His_6 TEV LIC ThurKC and pRSFDuet-1 MCS2 $ThurA_1$ constructs.[1]

Cloning, Expression and Purification of Modified $ThurA_1$ Peptide

The $ThurA_1$ insert was cloned from the genomic DNA of *Bacillus thuringiensis sv. andalousiensis* NRRL B23139 and ligated into a linearized pET28 MBP His_6 Thrombin-TEV vector (using a

Gibson Assembly Kit. The sequence-confirmed plasmid was co-transformed along with pET MBP LIC ThurKC into *E. coli* BL-21 Rosetta for production of the peptide. The MBP His_6-tagged $ThurA_1$ peptide was co-expressed with MBP-tagged ThurKC with ampicillin at 143 μM, kanamycin at 52 μM, and chloramphenicol at 62 μM as described and purified using a 5 ml Ni-NTA affinity column. Fractions were checked for purity using an SDS-PAGE where the MBP His_6 $ThurA_1$ co-eluted with the MBP-tagged ThurKC. The complex was concentrated using an 30kDa Amicon (Millipore Sigma) to a final concentration of 30 $g^{-1}L^{-1}$.

The concentrated complex was incubated for 20 hours at 18°C with TEV and 5 mM ATP and 5mM $MgCl_2$ to induce complete modification of the peptide. Upon cleavage of the MBP His_6 tag, a Ser-Gly-Ser was retained at the N-terminus of the peptide. After tag cleavage, 1 eq. of 50% acetonitrile (ACN) and 0.1% trifluoroacetic acid (TFA) was added to the reaction to precipitate MBP, TEV, and MBP-ThurKC. After centrifugation to remove the insoluble proteins, the supernatant was purified by HPLC using a VP NUCLEODUR 250 x10 mm, 5 μm, C8 column and a Shimadzu LC-20AD HPLC system. Chromatographic separation utilized a linear gradient (solvent A composed of H_2O and 0.1% Trifluoroacetic acid and solvent B composed of ACN and 0.1% TFA), starting at 30% B and going to 65% B over 40 minutes. Fractions were monitored by absorbance at 214 or 280 nm, and collected fractions were mixed in a 1:1 ratio with 2,5-dihydroxybenzoic acid matrix, spotted on a Bruker MALDI plate, and analyzed by MALDI-TOF-MS in a Bruker Daltonics UltrafleXtreme MALDI TOF/TOF instrument (Bruker). Fully modified $ThurA_1$ peptides, corresponding to 11 dehydrations and intermediates were identified by MALDI-TOF-MS and lyophilized for further use. Similar methods were used for the purification of modified $ThurA_7$ peptide.

Cloning, Expression and Purification of the $ThurA_1$ Leader Peptide

The $ThurA_1$ leader sequence was cloned from the genomic DNA of *Bacillus thuringiensis sv. andalousiensis* NRRL B23139 and ligated into a linearized the pET His_6 MBP TEV vector. The sequence-confirmed plasmid was plasmid was transformed into *E. coli* BL21 Rosetta for heterologous production as above with kanamycin at 52 μM and chloramphenicol at 62 μM. Following IPTG induction, cells were harvested by centrifugation (3500 rpm, 40 min, 4 °C) and resuspended in denaturing suspension buffer (8M Urea, 50 mM Tris pH 8.0, 150 mM NaCl). Cells

were lysed by sonication with 30 second cycles at 40% amplitude with 1 min resting intervals, for a total of seven cycles. The crude lysate was centrifuged for 60 minutes at 14000 rpm and 4 °C to clear insoluble cell debris. The pET His_6 MBP tagged $ThurA_1$ leader peptide was purified using a 5 ml Ni-NTA affinity column under denaturing conditions. Purified fractions were concentrated to 30 $g^{-1}L^{-1}$ using a 30 kDa Amicon (Millipore Sigma) and dialyzed overnight into a buffer of 20 mM Tris pH 8.0 and 150 mM NaCl. The dialyzed fraction was incubated for 20 hours at 18°C with TEV to remove the pET His_6 MBP tag. Cleavage of the tag retained a Ser-Asn-Ala linker at the N-terminus of the peptide.

After affinity tag cleavage, 1 eq. of 50% ACN and 0.1% TFA was added to the reaction to precipitate MBP and TEV. After centrifugation to pellet the insoluble proteins, the supernatant was purified by HPLC using a VisionHT C18 HighLoad 250 x 10mm, 5µm, C18 column and a Shimadzu LC-20AD HPLC system. Chromatographic separation utilized a linear gradient (solvent A composed of H_2O and 0.1% TFA and solvent B composed of ACN and 0.1% TFA), starting at 5% B and going to 65% B over 40 minutes. Fractions were monitored by absorbance at 214 nm, and collected fractions were mixed in a 1:1 ratio with 2,5-dihydroxybenzoic acid matrix, spotted on a Bruker MALDI plate, and analyzed by MALDI-TOF-MS in a Bruker Daltonics UltrafleXtreme MALDI TOF/TOF instrument (Bruker). Purified $ThurA_1$ leader peptide was lyophilized for crystallographic studies.

Cloning, Expression and Purification of Unmodified and Modified $\Delta C_8 ThurA_1$ peptide

Attempts to purify full-length unmodified $ThurA_1$ precursor failed due to proteolysis in situ. Analysis of the fragmentation pattern of the resultant peptide suggest that the precursor peptide was cleaved at Val14 of the core and was missing the C-terminal 8 residues. A peptide encompassing all but the C-terminal 8 residues (hereafter $\Delta C_8 ThurA_1$) was cloned into pET SUMO His_6 TEV vector, and sequence-verified clones were transformed into *E. coli* BL21 Rosetta and grown in liquid culture with ampicillin at 143 µM and chloramphenicol at 62 µM. The unmodified $\Delta C_8 ThurA_1$ peptide was purified under denaturing conditions, as described for the $ThurA_1$ leader peptide, and lyophilized.

For production of ThurKC modified $\Delta C_8 ThurA_1$, the ThurKC sequence was cloned into the

pAYCDuet-1 vector for co-expression. The pACYCDuet-1 ThurKC and pET Sumo His_6 TEV $\Delta C_8 ThurA_1$ plasmids were co-transformed into *E. coli* BL21 cells for protein expression and purification as described above.

Cloning, Expression and Purification of the leader proteases ThurP1/P2 and ThurP3/P4

The sequence of ThurP1/P2 and ThurP3/P4 share sequence identity to other proteases that are implicated in leader peptide removal.[2] The two sets of proteases were each cloned from genomic DNA of *Bacillus thuringiensis sv. andalousiensis* NRRL B23139 as a single insert in the vector a pET His_6 TEV LIC (gift from Scott Gradia, Addgene plasmid # 29656), with ThurP3 fused to a His tag. E. *coli* DH10β was used for transformation and plasmid production. The insertions were verified by DNA sequencing. The confirmed plasmid was transformed into *E. coli* BL21 Rosetta using LB plates with ampicillin at 143 μM and chloramphenicol at 62 μM. The protease complexes were expressed and purified using the same protocol and buffers as Ni-NTA purification method of pET His_6 LIC ThurKC. Protease complex purification was confirmed by SDS-PAGE gel and the fractions containing the protease complex were dialyzed overnight at 4 °C into a buffer with 300 mM NaCl, 20 mM Tris pH 8.0, and 10% glycerol. Following dialysis, ThurP1/P2 and ThurP3/4 was concentrated down to 5 mg/mL using a 30kDa Amicon concentrator (Millipore Sigma), aliquoted, and frozen for later use.

Leader removal of $ThurA_1$, $ThurA_7$, and ΔC8 $ThurA_1$ peptides and core purification

The modified $ThurA_1$ was resuspended in a cleavage buffer (150 mM NaCl and 20 mM Tris pH 8.0) and protease complex was added in a ratio of peptide to protease of 1: 100, and the peptide/ protease mixture was incubated for 24 hours at 18 °C with either ThurP1/P2 or ThurP3/P4. After leader removal from the was confirmed using the ThurP3/P4 only by MALDI-TOF-MS, 1 eq. of 50% ACN and 0.1% TFA was added to the reaction to precipitate the proteases. After centrifugation to pellet the insoluble protein, the supernatant was purified by HPLC using a VisionHT C18 HighLoad 250 x 10mm, 5μm, C18 column and a Shimadzu LC-20AD HPLC system. Chromatographic separation utilized a linear gradient (solvent A composed of H_2O and 0.1% TFA and solvent B composed of ACN and 0.1% TFA), starting at 30% B and going to 65% B over 40 minutes. Fractions were monitored by absorbance at 280 nm, and collected fractions were mixed in a 1:1 ratio with 2,5-dihydroxybenzoic acid matrix, spotted on a Bruker MALDI

plate, and analyzed by MALDI-TOF-MS in a Bruker Daltonics UltrafleXtreme MALDI TOF/TOF instrument (Bruker). Purified ThurKC modified $ThurA_1$ core peptide was identified and lyophilized for further use.

ThurP3/P4 was also active as the functional protease for the ThurKC modified $ThurA_7$ and was used in an identical manner to obtain the modified core. The same methods were used for HPLC purification of the $ThurA_7$ modified core as for $ThurA_1$. Interestingly, the ThurP3/P4 peptidases were not functional for the removal of the leader of the ThurKC modified ΔC_8ThurA_1. The ThurP1/P2 complex was efficient in leader removal of the ΔC_8ThurA_1 leader. The ThurKC modified ΔC_8ThurA_1 14 residue truncated core was isolated by HPLC using a VisionHT C18 HighLoad 250 x 10mm, 5µm, C18 column and a Shimadzu LC-20AD HPLC system. The chromatographic separation utilized a linear gradient (solvent A composed of H2O and 0.1% TFA and solvent B composed of ACN and 0.1% TFA), starting at 5% B and going to 60% B over 40 minutes.

$ThurA_1$ peptide in vivo co-expressions with mutant ThurKC

Primers used for cloning are listed in Table S2. All mutants were cloned using Site-directed, Ligase-Independent Mutagenesis (SLIM), with primers designed based on the pET MBP LIC ThurKC sequencing data. Peptide and protein expression is the same as that described in the previous section. Following the incubation of the eluted mutant ThurKC and MBP His_6 $ThurA_1$ overnight with TEV, the free modified $ThurA_1$ peptides were then clarified by centrifugation and desalted using C18 ZipTips (Millipore Sigma). The samples were analyzed by MALDI-TOF-MS for crude analysis of the peptide expression. Identical methodology was done on the mutants of $ThurA_1$ leader peptide leader and WT ThurKC.

Cloning and expression of ThurKC subdomains and analytical size exclusion chromatography.

The three domains of ThurKC were cloned using the primers listed in S2. The lyase domain, ranging from amino acid 1-211, was cloned into both pET His_6 TEV LIC and pET His_6 MBP TEV LIC cloning vectors, but both ThurKC lyase domain constructs resulted in insoluble, degraded fragments. The lyase-kinase domain, ranging from amino acids 1-486, was cloned into pET His_6

TEV LIC, which resulted in soluble protein expression. The kinase domain, ranging from amino acid 223-486, was cloned into both pET His_6 TEV LIC and pET His_6 MBP TEV LIC cloning vectors. The pET His_6 TEV LIC ThurKC kinase domain only resulted in soluble protein when co-expressed with pRSFDuet-1 MCS1 $ThurA_1$ precursor peptide, and the MBP-kinase domain construct yielded soluble protein but precipitated upon tag removal. The cyclase domain, ranging from amino acid 487-872, was cloned into the MBP pET His_6 MBP TEV LIC cloning vector and the tag was cleaved successfully for assays.

Analytical size exclusion was run for the WT ThurKC, lyase-kinase, cyclase, and a 1:1 ratio mixture of the latter two was run on a Superdex 200 Increase 10/300 GL column in a buffer of 40 mM HEPES on 150 mM KCl at a flow rate of 0.5 mL/min in a GE AKTA 10 UPC FPLC system. Elution traces were plotted and analyzed using OriginPro.

Chemical modification of the Dha/Dhb residues

β-mercaptoethanol adduct formation was performed according to procedures previously described.[3] Andalusicin cores ($ThurA_1$ for A, $ThurA_7$ for B, 0.2 mg) was dissolved in the reaction mixture consisting of 280 µl ethanol, 200 µl water, 65 µl 5 M NaOH, and 60 µl β-mercaptoethanol and incubated at 50°C for 2 h. After the incubation was completed, the reaction mixture two-fold diluted with water and desalted using C18 ZipTips (Millipore Sigma) according to manufacturer's protocol, and subjected to MALDI-TOF-MS analysis.

Dehydrated $ThurA_1$ and $ThurA_7$ fragmentation spectra

$ThurA_1$ and $ThurA_7$ fully modified peptides were mixed 1:1 ratio with 2,5-dihydroxybenzoic acid matrix, spotted on a Bruker MALDI plate, and analyzed by MALDI-TOF-MS in a Bruker Daltonics UltrafleXtreme MALDI TOF/TOF instrument (Bruker). Spectra of fragmentation were obtained in LIFT mode; the accuracy of product ions measurement was within 1 Da range. Mass spectra were processed using FlexAnalysis 3.2 software and analyzed manually.

***In vitro* ThurKC Activity Assay**

The purified ΔC8$ThurA_1$ was resuspended in 150 mM NaCl and 20 mM Tris pH 8.0 to a concentration of 200 µM. The purified ThurKC from the pET His6 TEV LIC ThurKC and

pRSFDuet-1 MCS2 $ThurA_1$ co-expression construct was added to a 20 μM concentration, along with 5 mM and ATP and 5mM $MgCl_2$. The reaction was incubated for 5 hours at 25 °C and desalted using C18 ZipTips (Millipore Sigma). The samples were analyzed by MALDI-TOF-MS for analysis of the Dhb formation.

***In vitro* LP-ThurKC Activity Assay**

The $ThurA_1$ 9mer and $ThurA_1$ 14mer peptide core peptides were purchased from GenScript with 90% purity and were used directly. The purified LP-GS_7-ThurKC was added to a 20 μM concentration, along with 5 mM and ATP and 5mM $MgCl_2$. The reaction was incubated for 5 hours and desalted using C18 ZipTips (Millipore Sigma). The samples were analyzed by MALDI-TOF-MS for analysis of the Dhb formation.

Crystallization, Data Collection, Phasing and Refinement

Initial crystallization conditions for ThurKC incubated with 1:15 ratio to $ThurA_1$ leader peptide and 2 mM ATP and $MgCl_2$ were established using sitting drop sparse matrix screening. Preliminary conditions were optimized to a final condition consisting of 15% glycerol, 16% PEG 6000, 80 mM sodium cacodylate pH 6.5, 160 mM calcium acetate. Diffraction quality crystals were grown by the hanging drop method. Briefly, a 1 μL solution of 18 mg/mL ThurKC and leader peptide were mixed with 1 μL of the above precipitant and incubated over a solution of the same at 9 °C. Plate-like crystals grew over 7 days and were transiently soaked in the precipitant solution supplemented with 20% glycerol immediately prior to vitrification by direct immersion into liquid nitrogen.

The data set collected in which the leader peptide is not observed was collected from a crystal grown when 1 μL of ThurKC at 18 mg/mL was incubated with 1 μL of 18% (w/v) PEG 3350, 100 mM Bis tris propane/ hydrochloric acid pH 7.5, 200 mM sodium fluoride, and 3% 2-propanol. Plate-like crystals grew over 5 days and were transiently soaked in the precipitant solution supplemented with 25% glycerol immediately prior to vitrification into liquid nitrogen.

Attempts to obtain phases of cocrystals of ThurKC in complex with the $ThurA_1$ leader peptide did succeed, due to the low yields of this same protein construct in M9 media and inability to obtain

anomalous signal for multiple heavy atom-soaked crystals. We created the single chain fusion construct as described in an earlier section. The LP-ThurKC was incubated at 18 mg/mL with 3 mM ATP and 3 mM $MgCl_2$ and screened using sitting drop sparse matrix screening. Initial conditions were optimized to 0.2 M potassium citrate tribasic monohydrate and 19 % w/v PEG 3350. Diffraction quality crystals were grown by the sitting drop method. Briefly, a 1 μL solution of 23 mg/mL of LP-ThurKC, 2 mM ATP and 2 mM $MgCl_2$ (1 μL) was mixed with 1 μL of the latter precipitant and incubated over a solution of the same at 9 °C. Large plate crystals grew over 7 days, and were subsequently soaked for 6 hours at 9 °C in the precipitant solution supplemented with 7 mM ATP and 7mM $CaCl_2$. The crystals were then transiently soaked in precipitant solution supplemented with 20% glycerol immediately prior to vitrification by direct immersion in liquid nitrogen. Selenomethionine incorporated LP-ThurKC was similarly screened using a sparse matrix screen. The best diffracting crystals were obtained by incubation of the selenomethionine derived LP-ThurKC incubated with 2 mM ATP and 2 mM $MgCl_2$ on two different conditions: 1) 0.09 mixture of sodium nitrate, sodium phosphate dibasic, and ammonium sulfate, 0.1 M buffers Tris base and Bicine pH 8.5, and precipitant mix of 20% v/v PEG 500 MME, 10% w/v PEG 20,000; 2) 0.2 M ammonium sulfate, 0.1 M Tris pH 8.5, and 25% PEG 3350. The crystals were vitrified as above prior to data collection.

Diffraction data were collected at the Advanced Photon Source Sector-21 LS-CAT at Argonne National Labs at beamlines 21-ID-D, 21-ID-F, and 21-ID-G. All diffraction data were indexed, scaled, and integrated using autoPROC.[4] Crystallographic phases were determined for LP-ThurKC using the CRANK2[5] pipeline as implemented in CCP4[6] from data collected on SeMet labelled protein crystals. An initial model was produced using Parrot and Buccaneer,[7] and this was further improved through manual model building with COOT.[8] Phases for the datasets of ThurKC incubated with $ThurA_1$ leader were determined using Phaser,[9] using the LP-ThurKC structure as a search model. The models were subject to rounds of manual building followed by refinement using REFMAC5[10] and PHENIX.Refine[11] until convergence Crystallographic statistics are listed in Table S4.

Protein	Accession code
ThurKC	WP_172554310.1
$ThurA_1$	WP_172554311.1
$ThurA_3$	WP_172554312.1
$ThurA_7$	WP_172554313.1
ThurP1	WP_048545971.1
ThurP2	WP_172554144.1
ThurP3	WP_172554699.1
ThurP4	WP_172554700.1
AciKC	WP_015792832.1
LabKC	WP_182844162.1
EryKC	WP_009949110.1
StaKC	WP_013015817.1
AmyKC	WP_013351405.1
ApiKC	AHB63593.1
FlaKC	WP_012924048.1
MicKC	AVH76818.1

Table S1: NCBI accession numbers.

Primer Name	Sequence
ThurA1_LIC_F	TACTTCCAATCCAATGCAATGAATACAGTATTGGAATTACAAAAACTAGCG
ThurA1-Leader_LIC_F	TTATCCACTTCCAATGTTATTATGCTGCTTGACCCTTACCTTCTG
deltaC8_ThurA1_LIC_R	TTATCCACTTCCAATGTTATTAAACACCAGTTGTTGTTACTGTCCATGTTGTTGT
ThurA1_pET28_F	CTTCCAATCCGGATCCAATACAGTAGCGGAATTACAAAAACTAGCG
ThurA1_pET28_R	GAGTGCGGCCGCGACTTAGCAATTATTACTAATGGTTGATGCCCAAACAC
ThurA1_L5A_FLP	AATACAGTAGCGGAATTACAAAAACTAGCGCATGACACAGAAGGTAAGG
ThurA1_L5A_FP	AAAAACTAGCGCATGACACAGAAGGTAAGG
ThurA1_L5A_RLP	GTAATTCCGCTACTGTATTGGATCCGGATTGGAAGTACAGG
ThurA1_L5A_RP	GGATCCGGATTGGAAGTACAGG
ThurA1_L7A_FLP	GTATTGGAAGCGCAAAAACTAGCGCATGACACAGAAGGTAAGGGTCAAG
ThurA1_L7A_FP	CGCATGACACAGAAGGTAAGGGTCAAG
ThurA1_L7A_RLP	CTAGTTTTTGCGCTTCCAATACTGTATTGGATCCGGATTGGAAGTACAG
ThurA1_L7A_RP	TGTATTGGATCCGGATTGGAAGTACAG
ThurA1_Q8A_FLP	TTGGAATTAGCGAAACTAGCGCATGACACAGAAGGTAAGGGTCAAGC
ThurA1_Q8A_FP	CATGACACAGAAGGTAAGGGTCAAGC
ThurA1_Q8A_RLP	GCGCTAGTTTCGCTAATTCCAATACTGTATTGGATCCGGATTGGAAGTAC
ThurA1_Q8A_RP	TACTGTATTGGATCCGGATTGGAAGTAC
ThurA1_L10A_FLP	TACAAAAAGCGGCGCATGACACAGAAGGTAAGGGTCAAGCAGC
ThurA1_L10A_FP	ACAGAAGGTAAGGGTCAAGCAGC
ThurA1_L10A_RLP	GTCATGCGCCGCTTTTTGTAATTCCAATACTGTATTGGATCCGGATTGG
ThurA1_L10A_RP	ATTCCAATACTGTATTGGATCCGGATTGG
ThuP3_LIC_F	TACTTCCAATCCAATGCAATGGAAGATACATATATAAGTTCAAGTGAACAAAACGGATTACAG
ThuP4_LIC_R	TTATCCACTTCCAATGTTATTATTATTCTCCTTTTACAATTAGTGTTGTTCTAGATTCAT
ThuP1_LIC_F	TACTTCCAATCCAATGCAATGAAACTAATGGAACAACAGTTACATGAGTTAGGTGG
ThuP2_LIC_R	TTATCCACTTCCAATGTTATTATTATTTTTTCGGTAAAACTTGACAAACACTCATTTTCT
ThurKC_LIC_F	TACTTCCAATCCAATGCAATGGAAGGAAATATGCTTTATCATCGGTATTTG
ThurKC_LIC_R	TTATCCACTTCCAATGTTATTAAATTTTCCCACTAGTTACTGATAAATCCTTGACTTTTG
ThurKC_pET22_F	CTGGAGGATCCCGAAGGAAATATGCTTTATCATCGGTATTTGAAACC
ThurKC_pET22_R	GGTGGTGCTCGAGTTATTAAATTTTCCCACTAGTTACTGATAAATCCTTGAC
ThurKC_pACYCDuet_F	CATCACCACAGCCAGATGGAAGGAAATATGCTTTATCATCGGTATTTG
ThurKC_pACYCDuet_R	CCGAGCTCGAATTCGTTAAATTTTCCCACTAGTTACTGATAAATCCTTGACTTTTG
LIC_ThurKC_plasmid_R	TTCATGGTGATGGTGATGGTGAGAAGATTTC
LIC_ThurKC_plasmid_F	ATGGAAGGAAATATGCTTTATCATCGGTATTTGAAACC
insert_A1L-GS7_F	CATCACCATCACCATGAAAACCTGTACTTCCAATCCAATGCAATGAATACAGTATTGG
insert_A1L-GS7_R	CATATTTCCTTCCATGGAACCGGACCCCGAGCCGCTTTCTGCTGCTTGACCCTTACCTTC
ThurKC_K65A_FP	AACTTCTTCATTAGAAGATTCAAAGGATGTACTAG
ThurKC_K65A_RP	GTTCTGGGAGTGTTGATCCTTTAACATGATAG
ThurKC_K65A_FLP	AGGGATGGGCAATTCATGTAACTTCTTCATTAGAAGATTCAAAGGATGTACTAG
ThurKC_K65A_RLP	ACATGAATTGCCCATCCCTGTTCTGGGAGTGTTGATCCTTTAACATGATAG
ThurKC_R163A_FP	GGATTTAAAGGTATATTTAATGAACATGGCGAGCATTG
ThurKC_R163A_RP	ATTGCTATTTTTCCATCTCTTATCATTAAGAATATAAGGTCC
ThurKC_R163A_FLP	GTATTTTATGCATATGGCGGATTTAAAGGTATATTTAATGAACATGGCGAGCATTG
ThurKC_R163A_RLP	GCCATATGCATAAAATACATTGCTATTTTTCCATCTCTTATCATTAAGAATATAAGGTCC

ThurKC_k117A_FP	ATATATCCTACGAATAACGAAGTCTTTGTTGAATTG
ThurKC_k117A_RLP	TGTTATAAACGCCCCGGAAGAAGCACGATTAGCATTTTTCGAATTCATCTTC
ThurKC_k117A_FLP	TCTTCCGGGGCGTTTATAACAATATATCCTACGAATAACGAAGTCTTTGTTGAATTG
ThurKC_k117A_RP	AGCACGATTAGCATTTTTCGAATTCATCTTC
ThurKC_R191A_FP	TATCAGGTGCCGGACTTTGTGAAAG
ThurKC_R191A_RLP	AAAGGGATTCGCTTGATCCTTAATTAAATTACCTTCCTTATCCCTAATACAATGCTCG
ThurKC_R191A_FLP	AAGGATCAAGCGAATCCCTTTTATCAGGTGCCGGACTTTGTGAAAG
ThurKC_R191A_RP	AATTAAATTACCTTCCTTATCCCTAATACAATGCTCG
ThurKC_H67A_FP	TTCATTAGAAGATTCAAAGGATGTACTAGATAAGG
ThurKC_H67A_RP	ATCCCTGTTCTGGGAGTGTTGATCCTTTAAC
ThurKC_H67A_FLP	GGAAAATTGCAGTAACTTCTTCATTAGAAGATTCAAAGGATGTACTAGATAAGG
ThurKC_H67A_RLP	GAAGTTACTGCAATTTTCCATCCCTGTTCTGGGAGTGTTGATCCTTTAAC
ThurKC_K94A_FP	GATAAAGATAGTTTTATGAAGATGAATTCGAAAAATGCTAATC
ThurKC_K94A_RP	GAATTCGCGCACCTTAAGGATAAAGATAGTTTTATGAAGATGAATTCGAAAAATGCTAATC
ThurKC_K94A_FLP	AATCTTCTTATCTATACATAATCGTGCAACCTTATCTAG
ThurKC_K94A_RLP	CTTAAGGTGCGCGAATTCAATCTTCTTATCTATACATAATCGTGCAACCTTATCTAG
ThurKC_D152A_FP	AAAATAGCAATGTATTTTATAGATATGGCGGATTTAAAGG
ThurKC_D152A_RLP	TCCATCTCTTCGCATTAAGAATATAAGGTCCTTTTTTAAAGTCCTGAATTGCTAAAG
ThurKC_D152A_FLP	TTCTTAATGCGAAGAGATGGAAAAATAGCAATGTATTTTATAGATATGGCGGATTTAAAGG
ThurKC_D152A_RP	TATAAGGTCCTTTTTTAAAGTCCTGAATTGCTAAAG
ThurKC_D382A_FP	TGCTATGCCTGTAAACTCTGATGACAGACCTGC
ThurKC_D382A_RP	CCTAACGGTTAAGTCCTCGGTTACCATTATATTTGCTG
ThurKC_D382A_FLP	ATAATAGCGTTTGAAACTGCTATGCCTGTAAACTCTGATGACAGACCTGC
ThurKC_D382A_RLP	GTTTCAAACGCTATTATCCTAACGGTTAAGTCCTCGGTTACCATTATATTTGCTG
ThurKC_R275A_FP	GAGTATGATGCTTTGAAAAAGCTTAAAGATGTATCTG
ThurKC_R275A_RP	CATCTTGAGCTGCTCCATCTAAACCAG
ThurKC_R275A_FLP	CATTAGCAGCGCAAAAAATCGAGTATGATGCTTTGAAAAAGCTTAAAGATGTATCTG
ThurKC_R275A_RLP	GATTTTTTGCGCTGCTAATGCATCTTGAGCTGCTCCATCTAAACCAG
ThurKC_K108A_FLP	GAATTCGGCGAATGCTAATCGTGCTTCTTCCGGGAAGTTTATAACAATATATCC
ThurKC_K108A_FP	CGTGCTTCTTCCGGGAAGTTTATAACAATATATCC
ThurKC_K108A_RLP	ATTAGCATTCGCCGAATTCATCTTCATAAAACTATCTTTATCCTTAAGGTGCTTGAATTC
ThurKC_K108A_RP	ATCTTCATAAAACTATCTTTATCCTTAAGGTGCTTGAATTC
ThurKC_E279A_FP	ATGCTTTGAAAAAGCTTAAAGATGTATCTGGTGTTGTTAATT
ThurKC_E279A_RP	TTTTGCCTTGCTAATGCATCTTGAGCTGC
ThurKC_E279A_FLP	AATCGCGTATGATGCTTTGAAAAAGCTTAAAGATGTATCTGGTGTTGTTAATTT
ThurKC_E279A_RLP	CATACGCGATTTTTTGCCTTGCTAATGCATCTTGAGCTGC
ThurKC_K257A_FP	GACCAAGTGCTGGTTTAGATGGAGCAGC
ThurKC_K257A_RP	TTTTAAGTTATCTTTCTTTCGAGTCGCAAGGTATACCCC
ThurKC_K257A_FLP	GTAATTATTGCAGAGGCCAGACCAAGTGCTGGTTTAGATGGAGCAGC
ThurKC_K257A_RLP	GGCCTCTGCAATAATTACTTTTAAGTTATCTTTCTTTCGAGTCGCAAGGTATACCCC
ThurKC_D266A_FP	CTCAAGATGCATTAGCAAGGCAAAAAATCG
ThurKC_D266A_RP	GCACTTGGTCTGGCCTCTTTAATAATTAC
ThurKC_D266A_FLP	TGGTTTAGCCGGAGCAGCTCAAGATGCATTAGCAAGGCAAAAAATCG
ThurKC_S726A_FLP	CCATACCTTGCGGGAGGATCTATTGGAGTTGCTATCTCAATCTGGTTTTTAAA
ThurKC_S726A_FP	ATTGGAGTTGCTATCTCAATCTGGTTTTTAAA

ThurKC_S726A_RLP	GATCCTCCCGCAAGGTATGGCAATAGACGATTTTTATTATCGACTGTTTGTAGTAC
ThurKC_S726A_RP	CAATAGACGATTTTTATTATCGACTGTTTGTAGTAC
ThurKC_F770A_FP	CAGGTAGCTTCCTTTTAATCCCTTCTATGGT
ThurKC_F770A_RLP	CACCATCCGCTAAGCCGCCACTAATTGTACAACGCGTCTTTGATAATTT
ThurKC_F770A_FLP	GGCGGCTTAGCGGATGGTGCAGGTAGCTTCCTTTTAATCCCTTCTATGGT
ThurKC_F770A_RP	ACTAATTGTACAACGCGTCTTTGATAATTT
ThurKC_D771A_FP	GGTAGCTTCCTTTTAATCCCTTCTATGGTTAA
ThurKC_D771A_RLP	TGCACCCGCAAATAAGCCGCCACTAATTGTACAACGCGTC
ThurKC_D771A_FLP	CTTATTTGCGGGTGCAGGTAGCTTCCTTTTAATCCCTTCTATGGTTAA
ThurKC_D771A_RP	CCGCCACTAATTGTACAACGCGTC
ThurKC_S608A_FP	GGTCTTTCAGGGATAGGTTTATTCGTAATTAG
ThurKC_S608A_RLP	TGAGCGTATCGCAATATCAGTTTGATTGATATTATCTTTTAAGATTTTCAACTCATTTAAGAC
ThurKC_S608A_FLP	CTGATATTGCGATACGCTCAGGTCTTTCAGGGATAGGTTTATTCGTAATTAG
ThurKC_S608A_RP	TTTGATTGATATTATCTTTTAAGATTTTCAACTCATTTAAGAC
ThurKC_D667A_FLP	GGTGTAATTGCGGGATTATCTGGTGTATCTTTGTTTTATTCGGCGC
ThurKC_D667A_FP	GGTGTATCTTTGTTTTATTCGGCGC
ThurKC_D667A_RLP	AGATAATCCCGCAATTACACCTATATCAACCGCCATCCAGTCCTTAACCTTTAACTGC
ThurKC_D667A_RP	TATATCAACCGCCATCCAGTCCTTAACCTTTAACTGC
ThurKC_Y826A_FP	TAGCTCTGGAATAATTTTAGCATTAATGGGAGTAATCAAAGGTAAT
ThurKC_Y826A_RP	ATCTGCCAATCTATAAGAGAATTGTCCTGGATATACATAGTAAC
ThurKC_Y826A_FLP	GATGTTGCTACAGGTAGCTCTGGAATAATTTTAGCATTAATGGGAGTAATCAAAGGTAAT
ThurKC_Y826A_RLP	CCTGTAGCAACATCATCTGCCAATCTATAAGAGAATTGTCCTGGATATACATAGTAAC
ThurKC_R519A_FP	TGAGATGAATGGGGGGAAATTTAACTTTTTGACTGG
ThurKC_R519A_RP	CCATTAATAAATCTTTCATCATTTGTTAAACTGCTTTCAACACC
ThurKC_R519A_FLP	TGATATTGCTCAGTTTGAGATGAATGGGGGGAAATTTAACTTTTTGACTGG
ThurKC_R519A_RLP	AACTGAGCAATATCACCATTAATAAATCTTTCATCATTTGTTAAACTGCTTTCAACACC
ThurKC_lyase_211end_RP	TTATCCACTTCCAATGTTATTAAATTGTATTAAGATAATCGTCGAAATCTTTCACAAAGT
ThurKC_kinase_223start_RP	TACTTCCAATCCAATGCAAGCAGATTAGGGAAATATAAGATTGAAACGGCGC
ThurKC_kinase_486end_RP	ATCCACTTCCAATGTTATTATTGATCATTAAGATTGATTTCTTTAGGAATAAATGTTTGG
ThurKC_cyclase_487start_FP	TACTTCCAATCCAATGCAACAAGTGATTTTAATTTAACTTCTATTATTAATAAATTAATA
ThurKC_lyase_D34start_FP	TACTTCCAATCCAATGCAGATATTCCTGATACATATGCTGTTTTCTTAGATAATGAATCA

Table S2: Primers synthetized for this study.

	LP-ThurKC Ca^{2+} ATP	$ThurA_1LP$ + ThurKC	ThurKC
**Data collection**			
Wavelength (Å)	1.00798	1.00385	1.12706
Resolution (Å)	2.15	2.50	2.52
Resolution Range	49.00 - 2.15<br>(2.16 - 2.15)	232.3 -2.5<br>(2.51-2.50)	115.12-2.52<br>(2.528-2.52)
Space group	$P2_1$	$P2_1$	$P2_1$
Cell dimensions			
a, *b*, *c* (Å)	54.0, 228.7, 84.6	81.0, 52.9, 233.4	55.3, 230.2, 84.4
β (°)	100.5	95.7	100.6
Total reflections	391098 (3809)	300289 (2043)	322334 (3368)
Unique reflections	94386 (928)	66801 (524)	68899 (698)
Multiplicity	4.1 (4.1)	4.5 (3.9)	4.7 (4.8)
Completeness (%)	86.6 (82.6)	96.9 (73.3)	98.9 (100.0)
$I / \sigma I$	9.1 (0.9)	9.8 (2.1)	12.4 (2.1)
R_{merge}^2 (%)	0.091 (1.372)	0.093 (0.519)	0.067 (0.824)
R_{meas} (%)	0.103 (1.555)	0.104 (0.603)	0.075 (0.925)
$CC_{1/2}$	0.998 (0.376)	0.995 (0.727)	0.998 (0.829)
**Refinement**			
Resolution (Å)	2.15	2.50	2.52
No. reflections	94214	63469	65420
R_{work} / R_{free}	0.202 /0.253	0.219 /0.321	0.186 / 0.252
No. atoms	14329	13872	13906
Protein	13860	13872	13906
Water	405	-	-
ATP/metals	64	-	-
B-factors			
Protein	60.74	64.54	90.40
Ligand	71.86	-	-
Water	52.00	-	-
R.m.s. deviations			
Bond lengths (Å)	0.009	0.009	0.004
Bond angles (°)	1.13	2.050	1.14

Table S3: Crystallographic Statistics for structures of ThurKC obtained in this study.

1. Highest resolution shell is shown in parenthesis.

2. R-factor = Σ(|Fobs|-k|Fcalc|)/Σ |Fobs|and R-free is the R value for a test set of reflections consisting of a random 5% of the diffraction data not used in refinement.

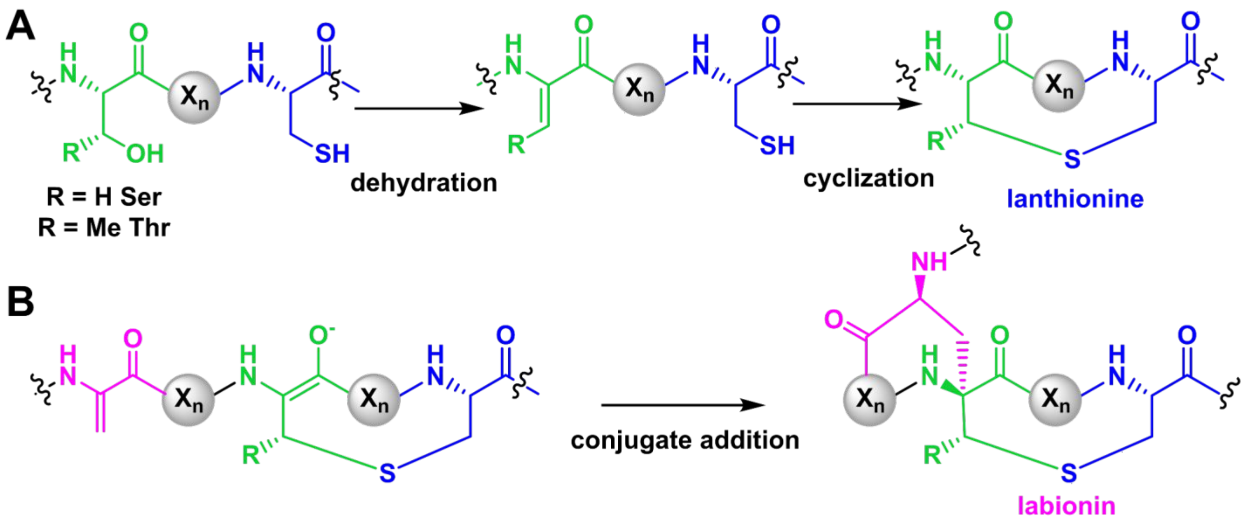

Figure S1: Biosynthetic scheme of labionin ring formation.

ThurA$_1$ MNTVLELQKLAHDTEGKGQAAEATITTTWTVTTTGVWASTISNNC
ThurA$_2$ MNTVLELQKLAHDTEGKGQAAEATITTTWTVTTTGVWASTISNNC
ThurA$_3$ MNTVLELQKLVHDTEGKGQAAEATITTTWTVTTTGVWASTISNNC
ThurA$_4$ MNTVLELQKLAHDTEGKGQAAEATITTTWTVTTTGVWASTISNNC
ThurA$_5$ MNTVLELQKLAHDTEGKGQAAEATITTTWTVTTTGVWASTISNNC
ThurA$_6$ MNTVLELQKLAHDTEGKGQAAEATITTTWTVTTTGVWASTISNNC
ThurA$_7$ MNTVLELQKLAHDTEGKGQAAEATITTTWTVTTTGAWASTISNNC

Figure S2: Precursor peptides encoded in andalusicin BGC. ThurA$_1$-ThurA$_6$ encode identical core peptide sequences. Hence, only ThurA$_1$ and ThurA$_7$ were used in this study.

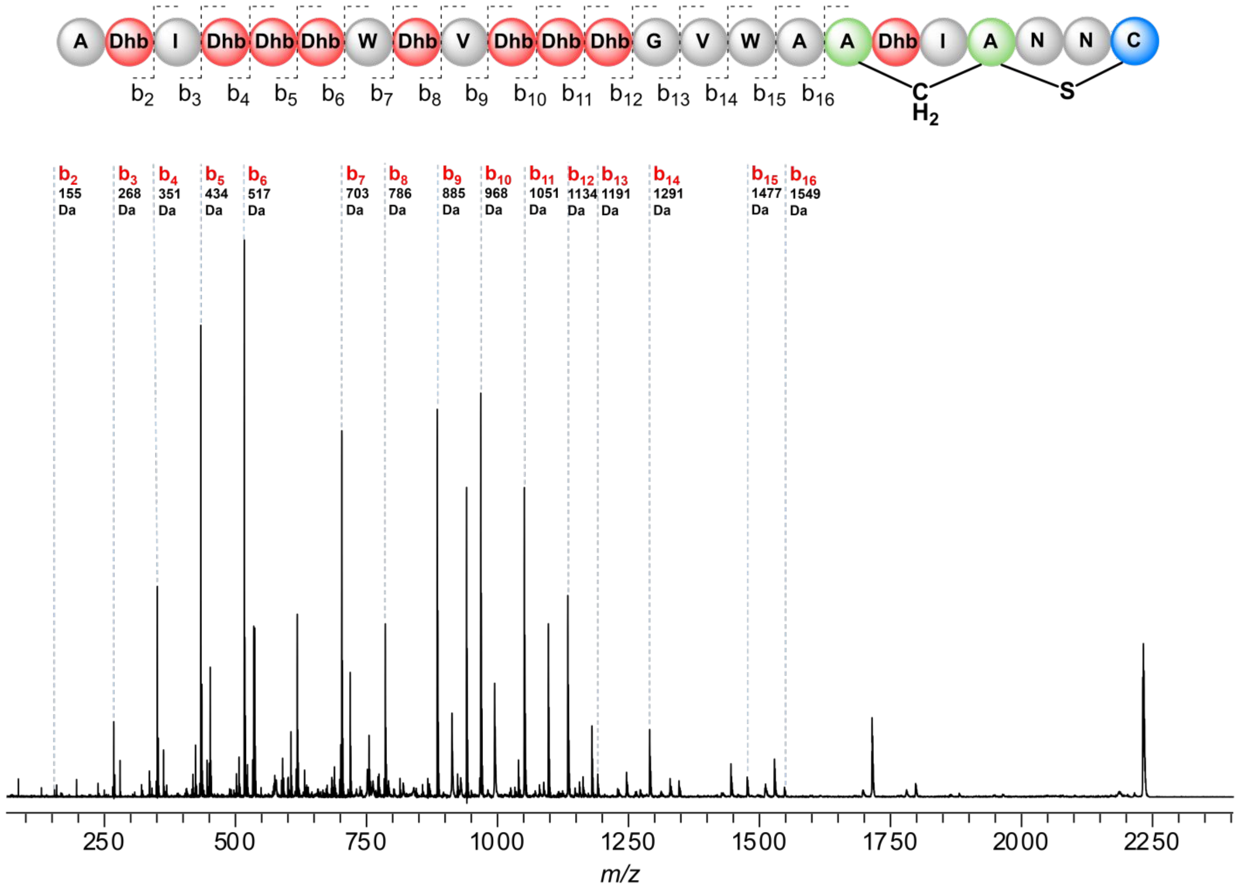

Figure S3: Tandem MS analysis of reconstituted ThurA$_1$. MALDI-TOF MS-MS analysis of mature ThurA$_1$ and structure proposed based on fragmentation pattern excluding residues 17-23.

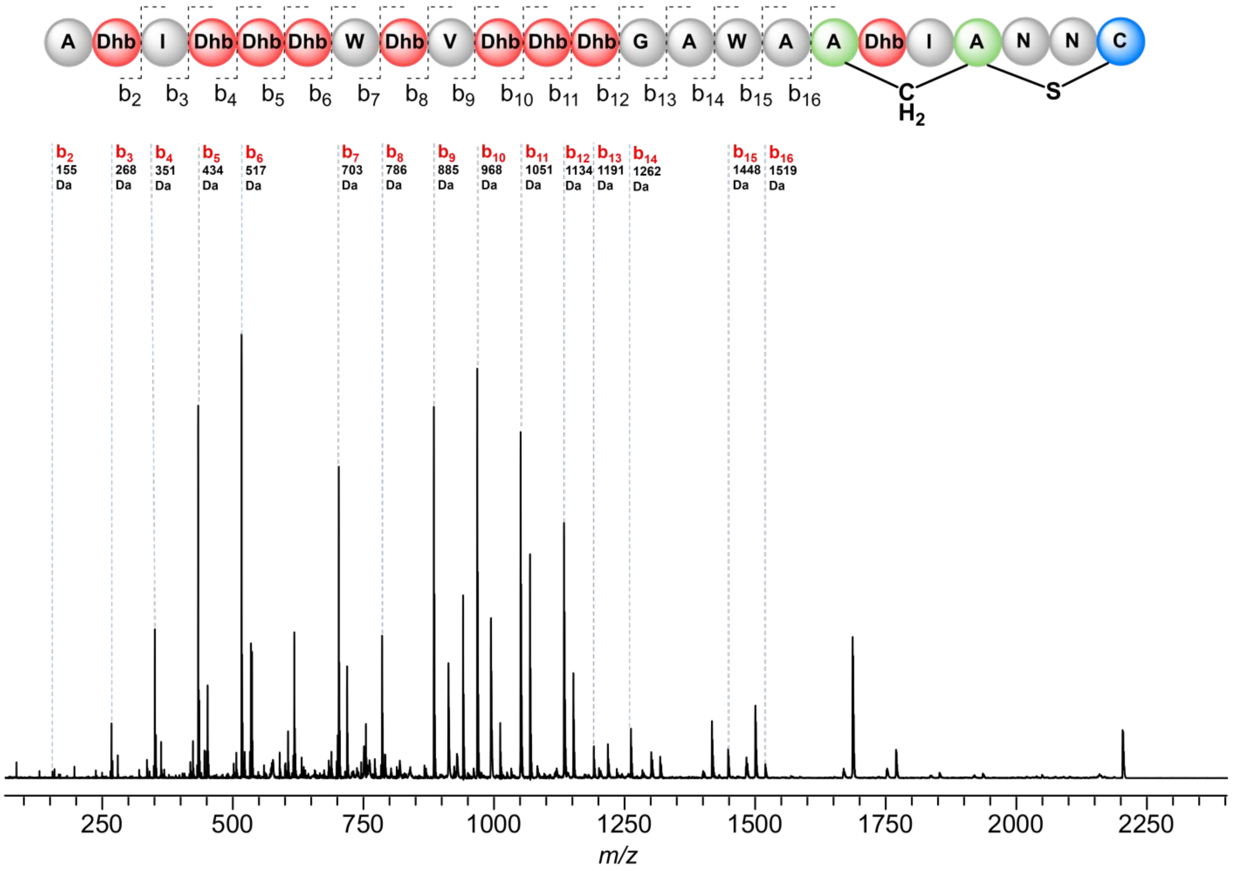

Figure S4: Tandem MS analysis of reconstituted ThurA$_7$. MALDI-TOF MS-MS analysis of mature ThurA$_7$ and structure proposed based on fragmentation pattern excluding residues 17-23.

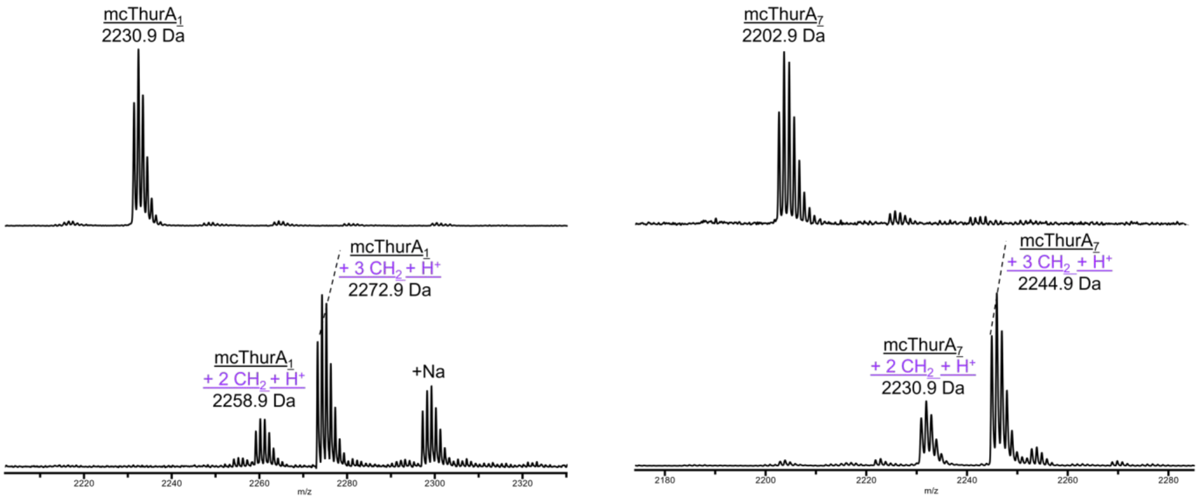

Figure S5: Incubation of modified core (mc) $ThurA_1$ or $ThurA_7$ after removal of their leader sequences with recombinant ThurMet methyltransferase and SAM yielded products with mass changes corresponding to the addition of two and three methyl groups.

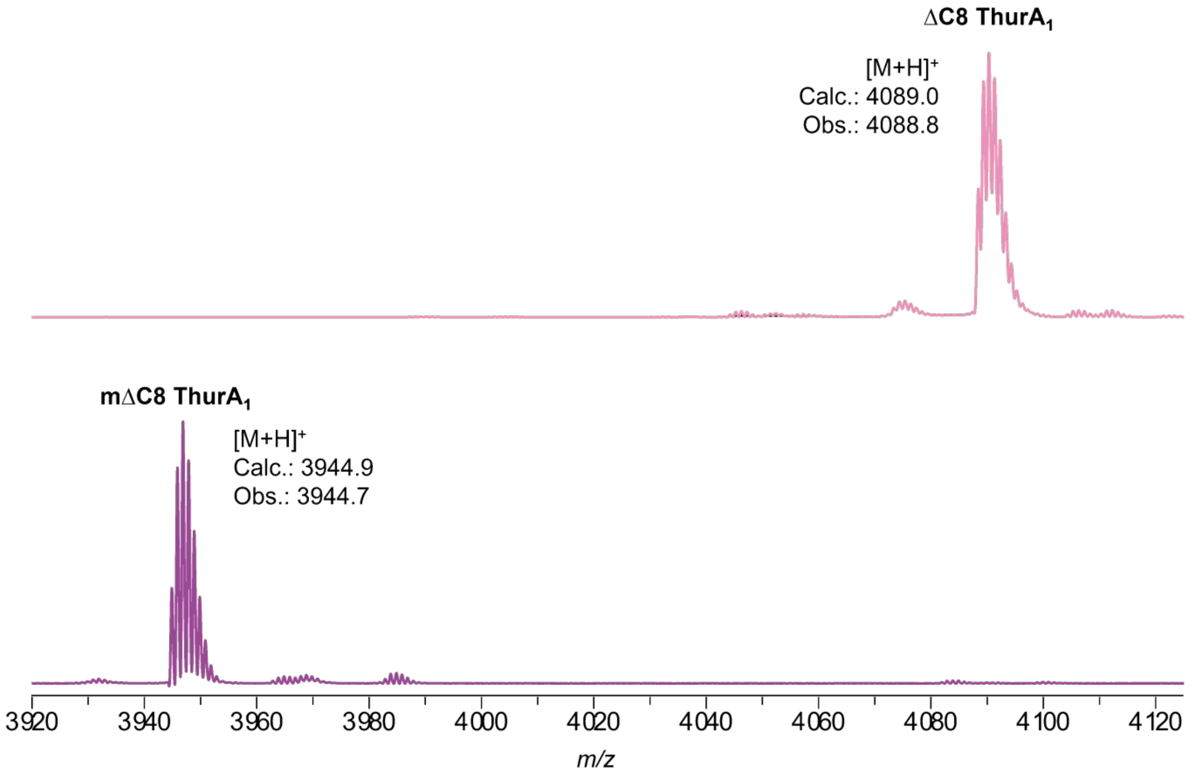

Figure S6: *In vitro* Dhb formation on ΔC8 $ThurA_1$ peptide following incubation with WT ThurKC, 5 mM ATP, and 5 mM $MgCl_2$.

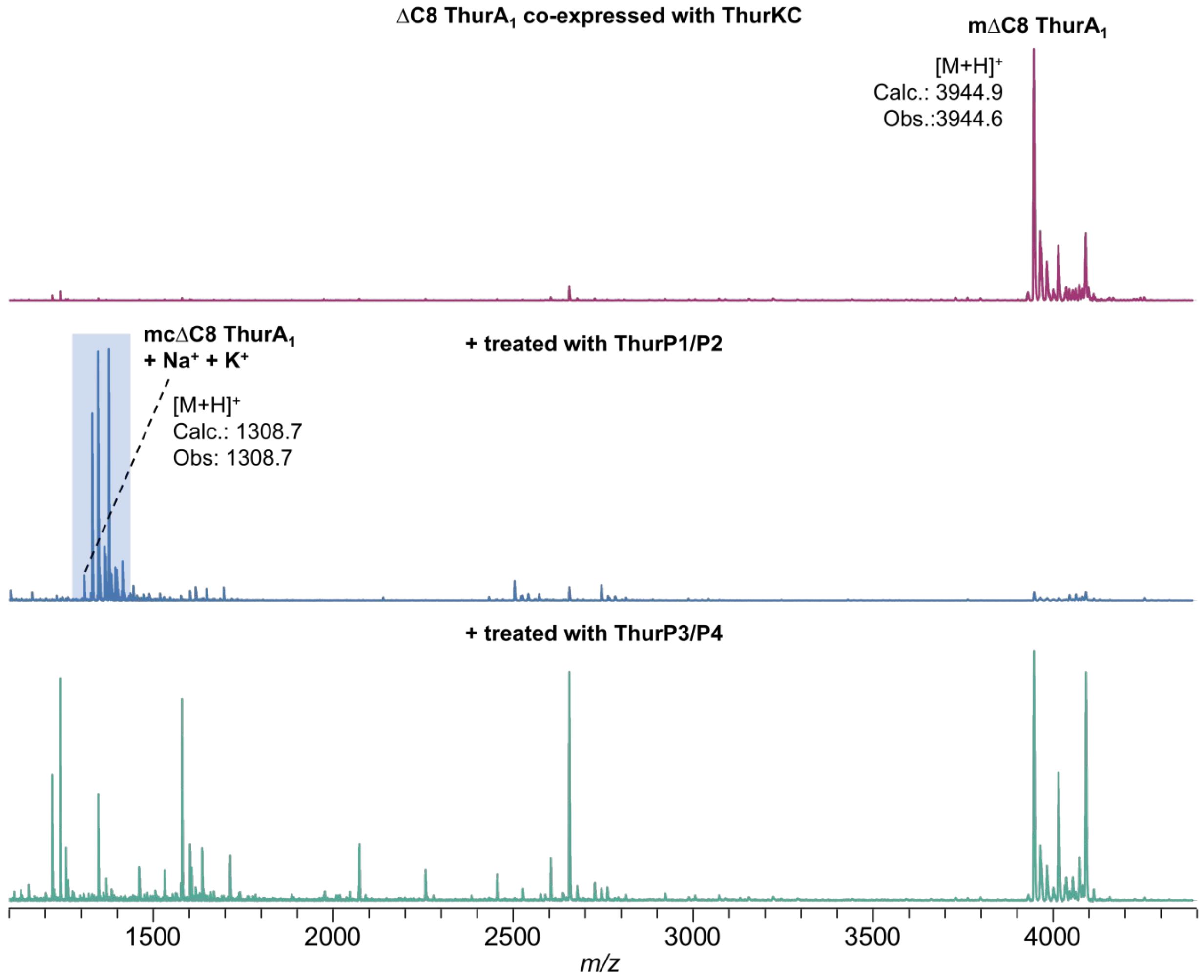

Figure S7: Heterologous expression of ThurKC modified ΔC8 (mΔC8) ThurA$_1$ in *E. coli* and leader removal of purified peptides analyzed by MALDI-TOF MS. Although the leaders of fully modified ThurA$_1$ and ThurA$_7$ peptides is removed efficiently by ThurP3/P4, the leader of the truncated mΔC8 ThurA$_1$ is most efficiently removed by ThurP1/P2 (see section marked "Cloning, Expression and Purification of the leader proteases ThurP1/P2 and ThurP3/P4").

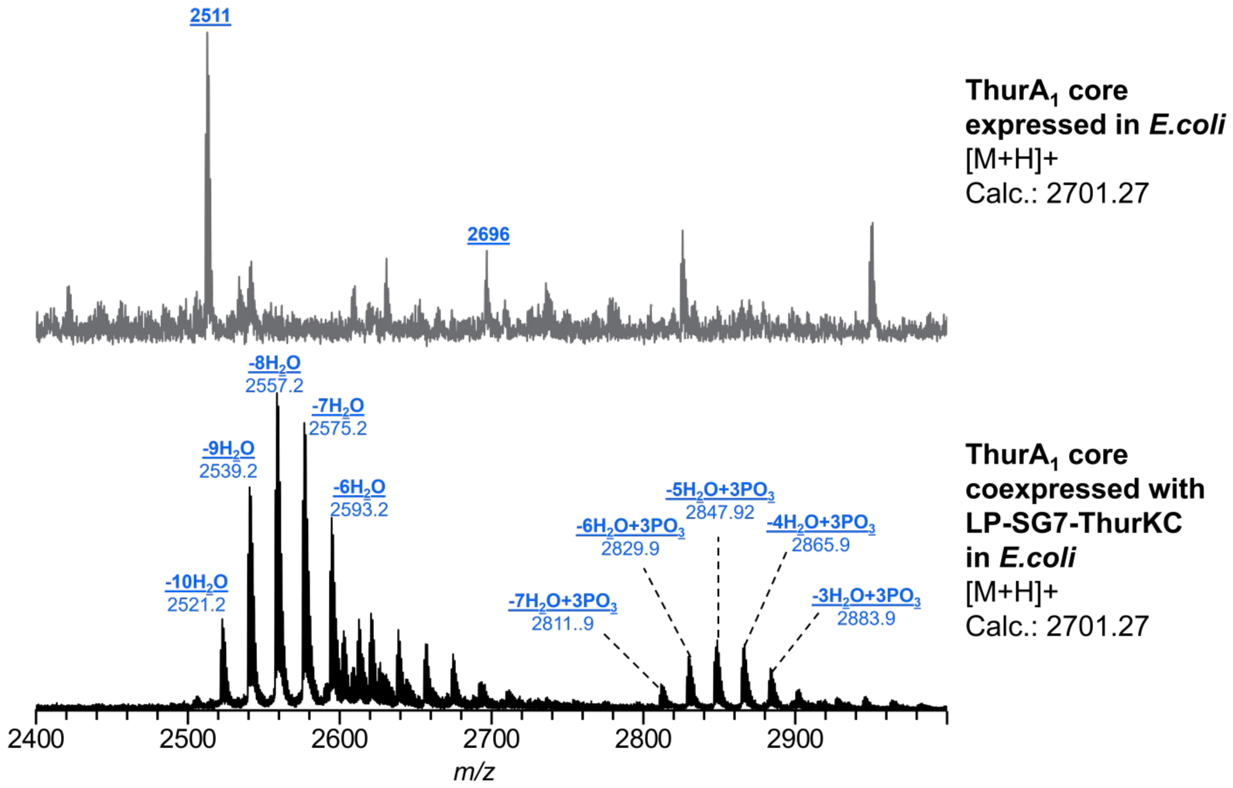

Figure S8: MALDI-TOF spectra of *in vivo* modified ThurA$_1$ core by WT ThurKC. The precursor ThurA$_1$ core (expected MW of 2701.27 Da) is degraded in E. coli when expressed with a His6-MBP N terminal tag, as revealed following tag removal with TEV. The same His6-MBP-TEV ThurA$_1$ core is modified and not degraded in vitro when expressed with the fusion LP-GS$_7$-ThurKC.

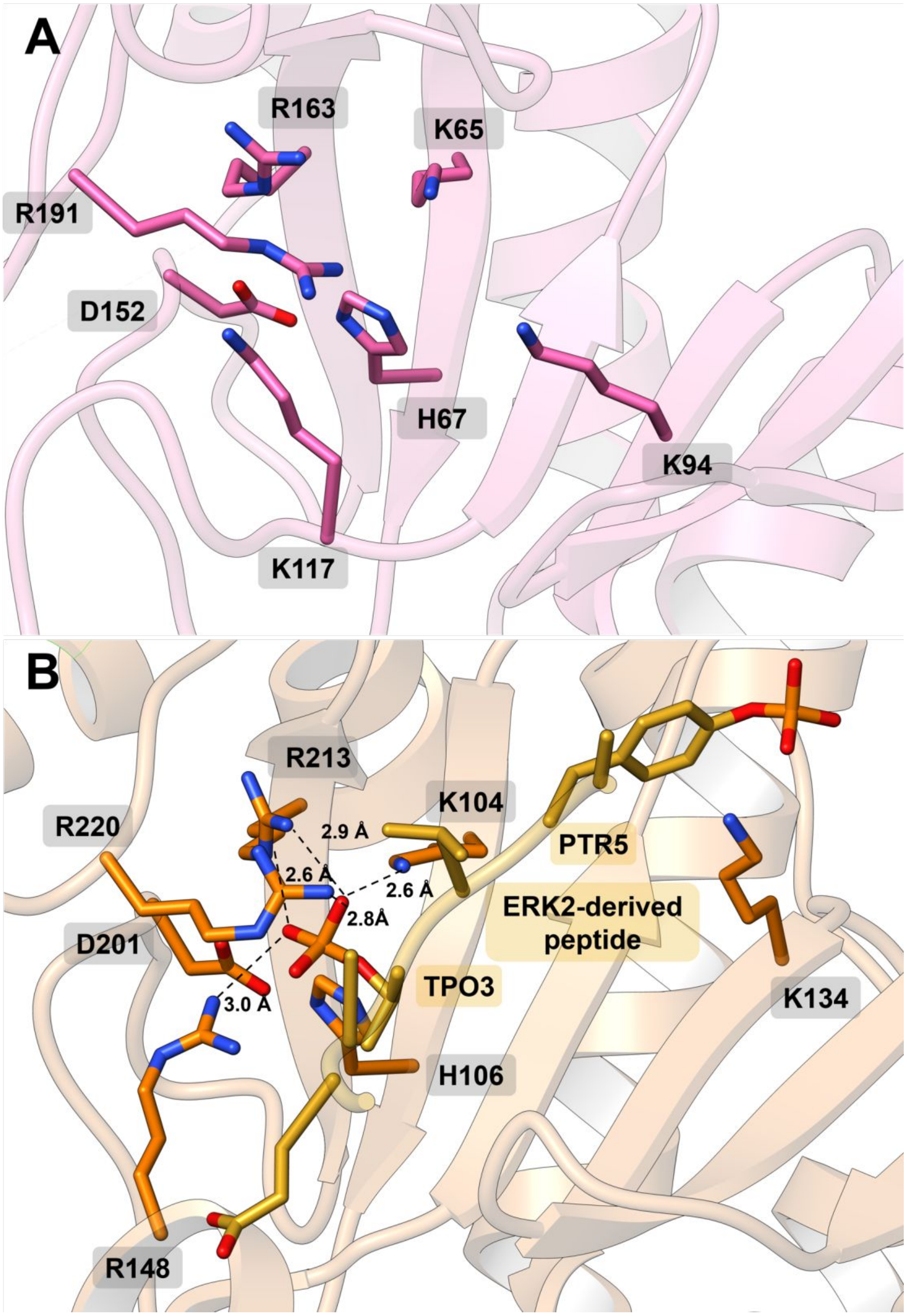

Figure S9: The ThurKC lyase domain in pink (top) compared with SpvC (in orange) bound to a phosphothreonine (TPO3) and phosphotyrosine (PTR5) modified peptide substrate derived from ERK2 (in gold) (PDB 2Z8P, bottom). Important conserved residues are shown as sticks.

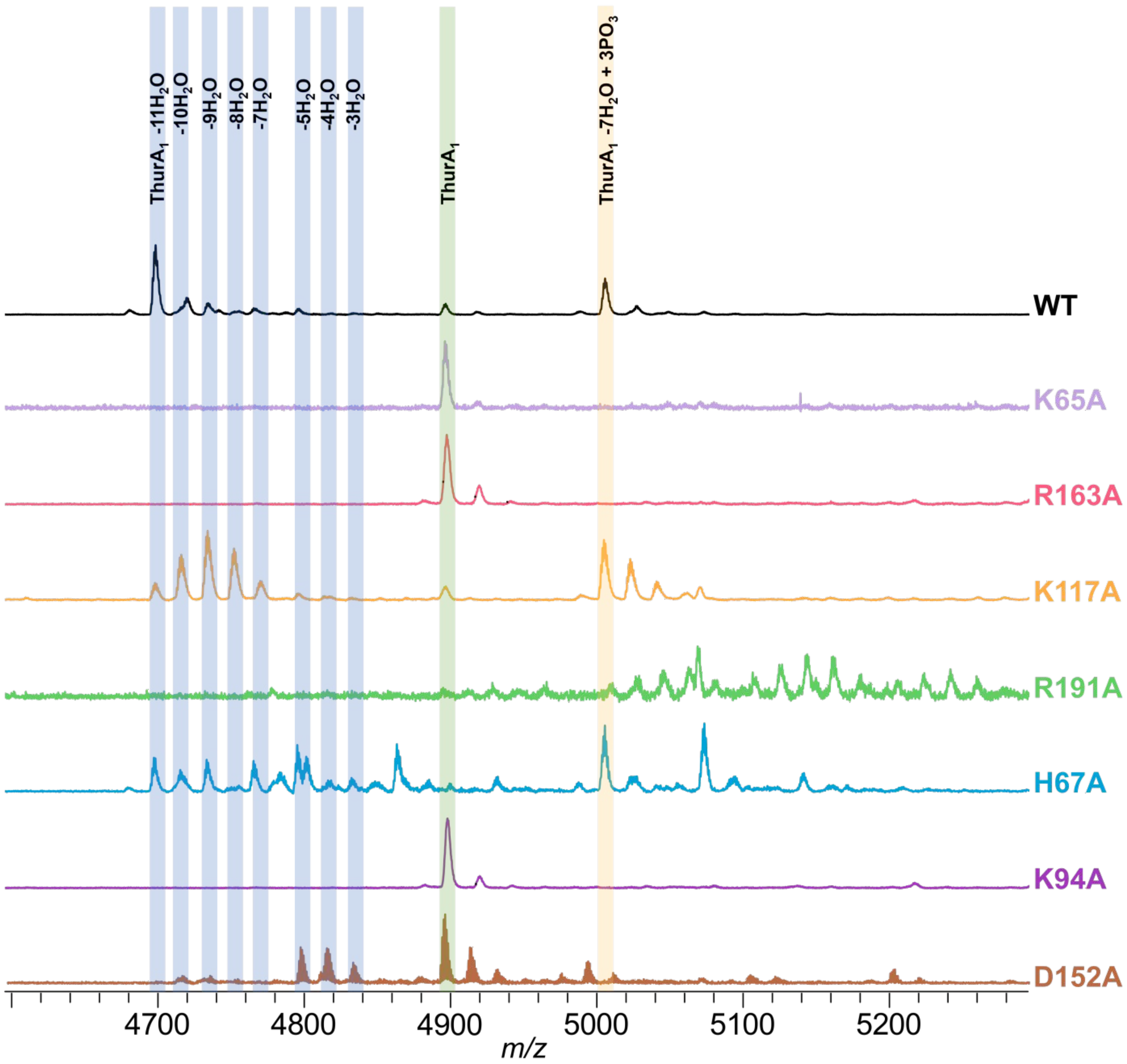

Figure S10: Heterologous co-expression of ThurA$_1$ peptide with ThurKC lyase domain variants in *E. coli* and analysis by MALDI-TOF MS.

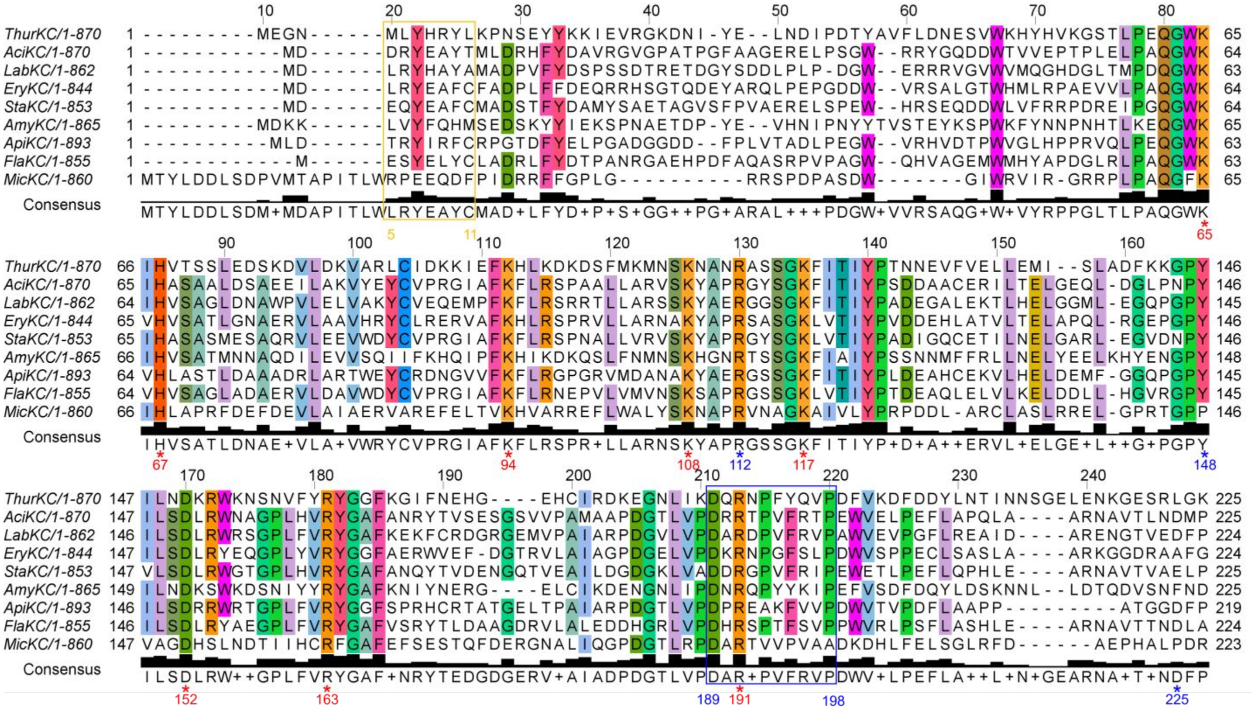

Figure S11: Sequence alignment of lyase domains of class III LanKC reported to produce lanthipeptide containing labionin. The alignment was performed by the Clustal Omega tool using the order of input. Above 50% conserved in sequence alignment is highlited and image is created using Jalview software. Residues predicted to be relevant to catalytic activity are marked with an red asterisk and corresponding residue number in ThurKC sequence. Residues and loops that are directly involved with binding the ThurA$_1$ leader and referred to in the main text are marked with an blue asterisk or box, respectively, and labeled with corresponding residue number in the ThurKC sequence. Residues corresponding to the ThurKC N-terminal helix are boxed in yellow.

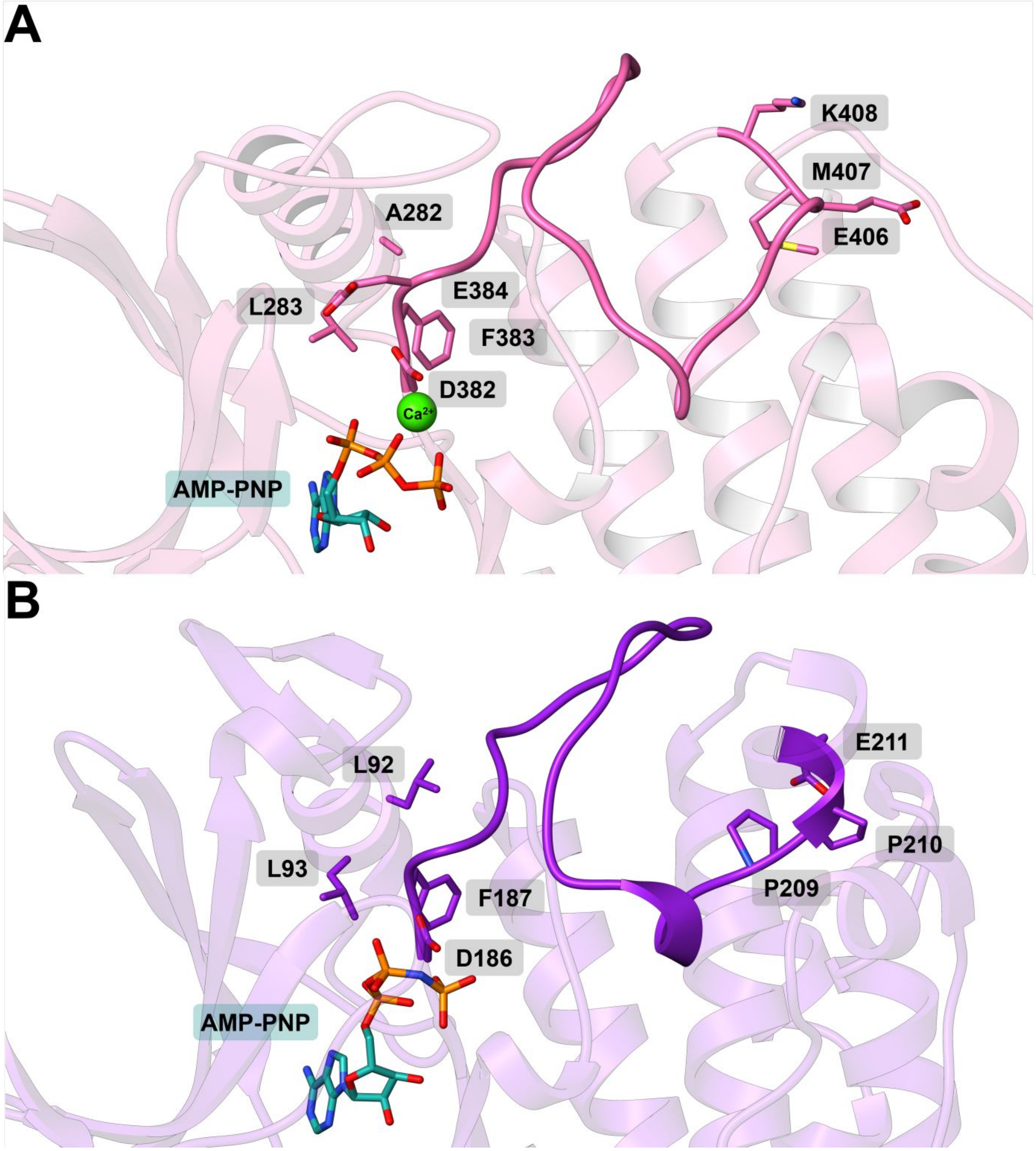

Figure S12: Comparison of the kinase domains of PIM-1 Kinase and ThurKC. (A) The ThurKC kinase domain (pink, residues shown as sticks) bound to ATP (turquoise) and Ca^{2+} ion (green) is compared with (B) PIM-1 kinase (purple, residues shown as sticks) bound to AMP-PNP (PDB 1YXT). The equivalent loop activation loop is highlighted, with DFE/DFG motif and APE motif residues shown as sticks.

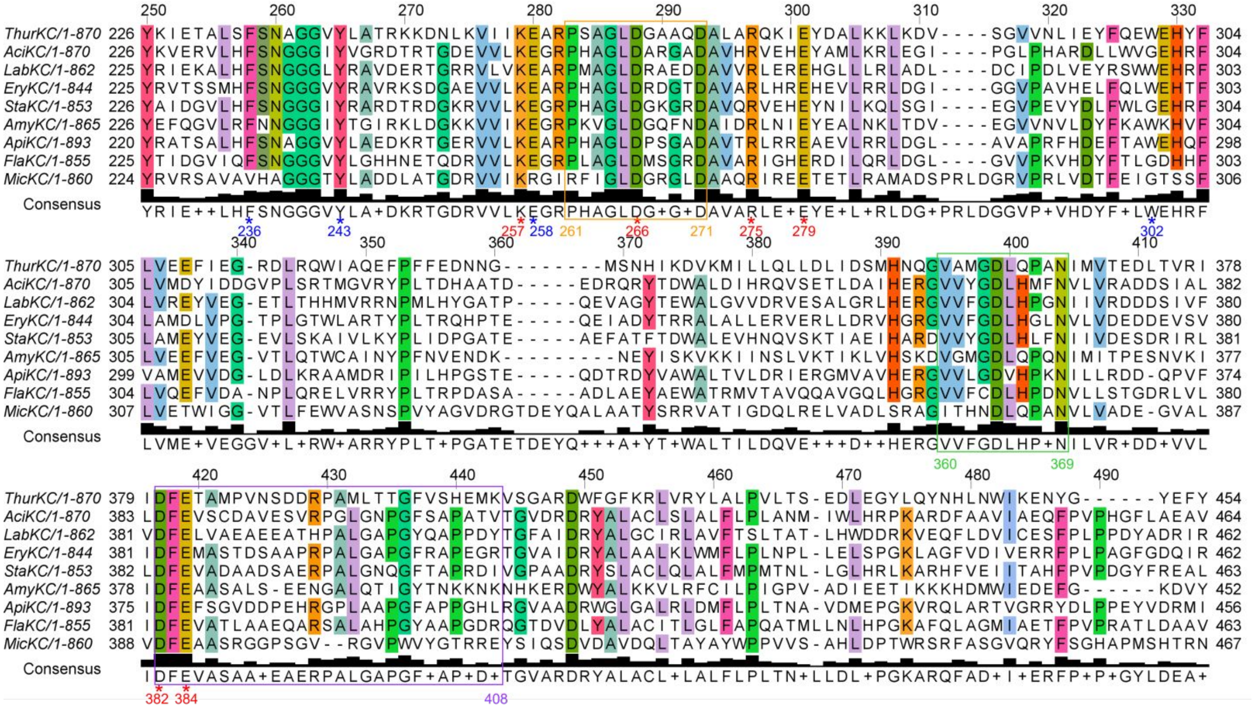

Figure S13: Sequence alignment of kinase domains of class III LanKC reported to produce lanthipeptide containing labionin. The alignment was performed by the Clustal Omega tool using the order of input. Above 50% conserved in sequence alignment is highlited and image is created using Jalview software. Above 50% conserved in sequence alignment is highlited. Conserved residues predicted to be relevant to catalytic activity are marked with an asterisk and corresponding residue number in ThurKC sequence. Residues that are directly involved with binding the ThurA$_1$ leader and referred to in the main text are marked with an blue asterisk and labeled with corresponding residue number in the ThurKC sequence. The activation loop, ranging from D382 to K408 is boxed in purple, the catalytic loop V360 to N369 is boxed in green, and the loop preceding the αC helix (P261 to D271) that aids in activation loop positioning is boxed in orange.

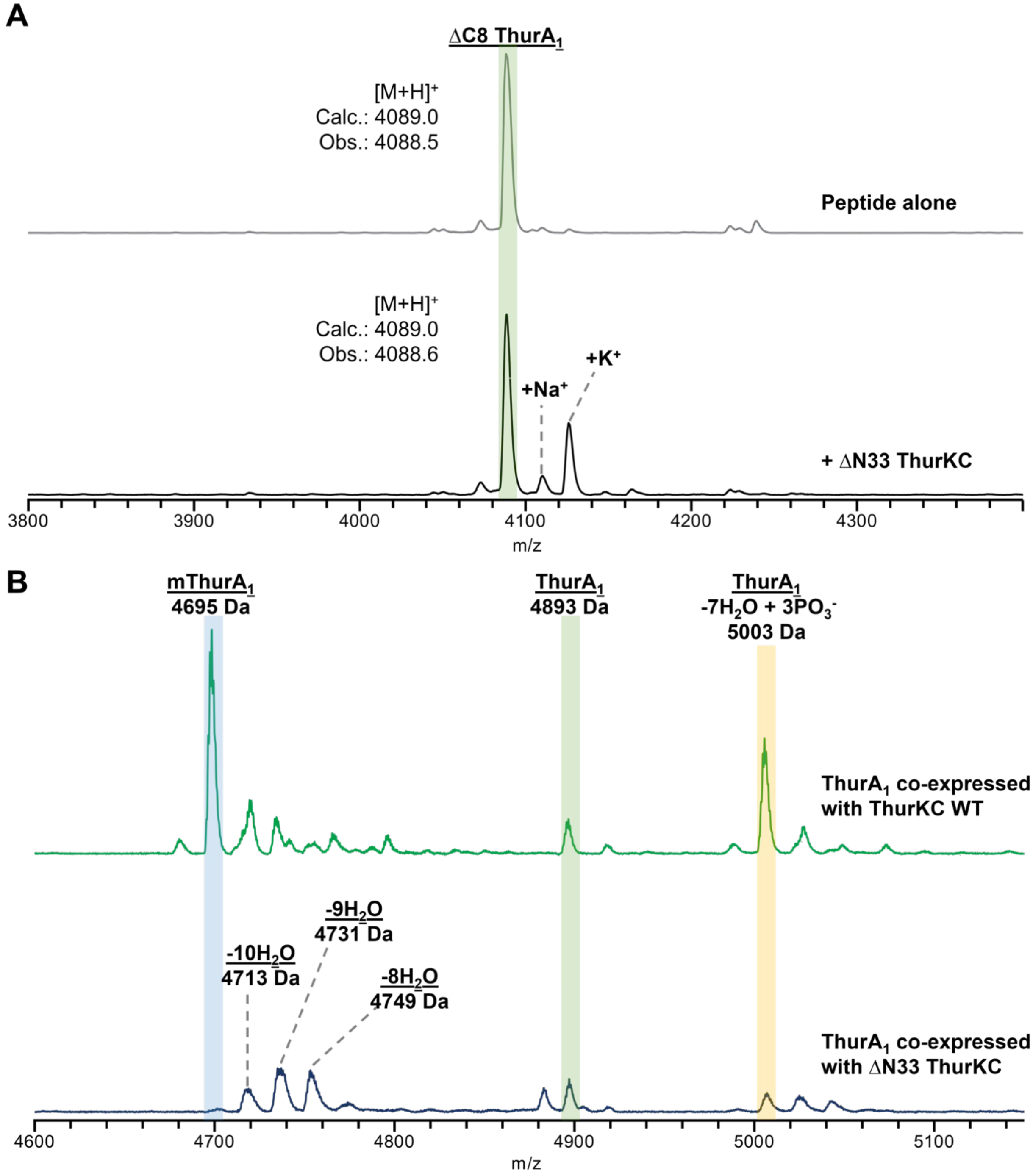

Figure S14: MALDI-TOF spectra ThurA$_1$ precursor peptides treated with ΔN33 ThurKC.

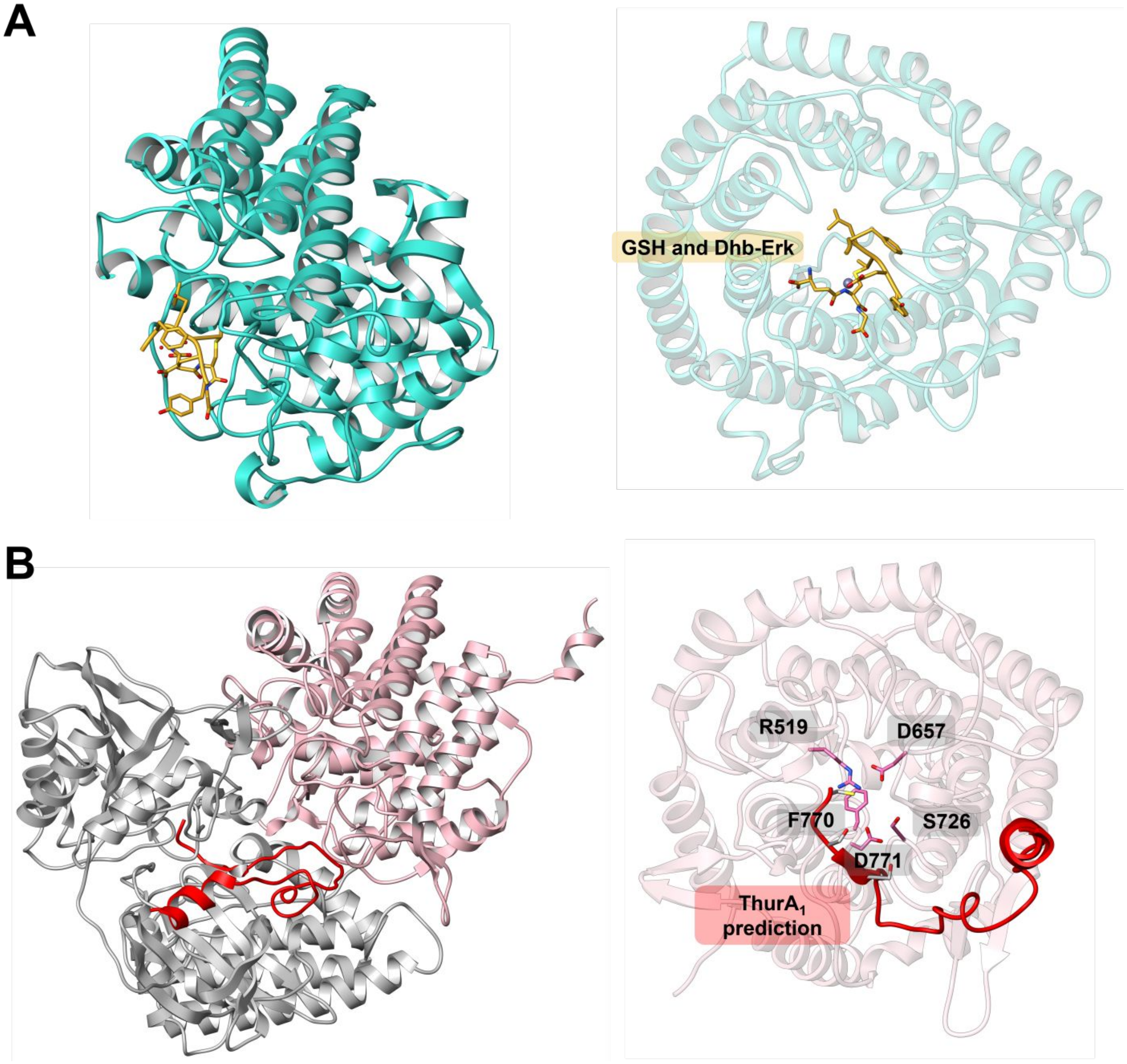

Figure S15: Comparison of the cyclase domains of LanCL1 and ThurKC. (A) Structure of the eukaryotic cyclase homolog LanCL1 bound to glutathionine and a substrate peptide derived from Erk. (B) An Alphafold model of ThurKC in complex with the full-length ThurA$_1$ peptide showing a close up view of the cyclase domain. Residues implicated in catalysis and tested biochemically are shown as stick figures.

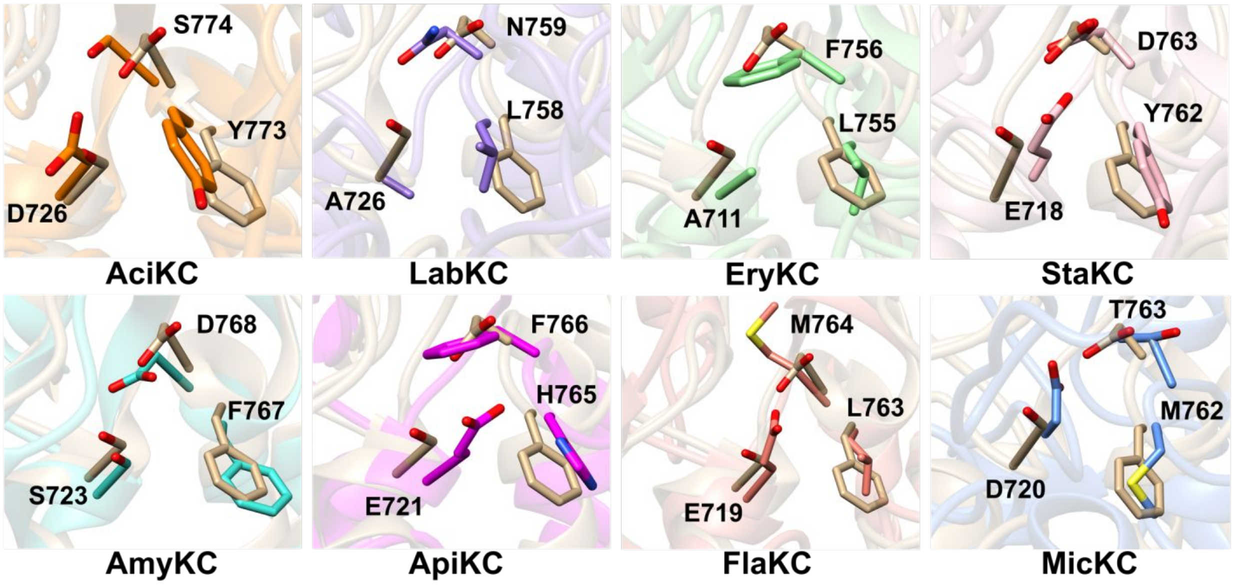

Figure S16: Alphafold active site predictions of LanKCs that have been biochemically characterized to produce Labionin rings. The classic zinc binding amino acid residues in NisC, Cys284, Cys330, and His331, are replaced by Ser726, Phe770, and Asp771 in ThurKC when the cyclase domains are superimposed. Alphafold is able to predict the stucture of ThurKC with a prediction confidence (predicted LDDT score) of 92.33, and positions the latter 3 residues in the same position as the LP-SG7-ThurKC X-ray structure bound to ATP and Ca^{2+}.

Alphafold predictions of LanKC enzymes were computed to observe whether ThurKC residues Ser726, Phe770, and Asp771 are conserved in the class III lanthionine enzymes reported to produce lanthipeptides with a labionin ring. Alphafold models AciKC (model score 90.92), LabKC (score 90.64), EryKC (score 92.94), StaKC (score 92.79), AmyKC (score 92.16), ApiKC (score 89.99), FlaKC (score 93.20), MicKC (87.91) were superposed with LP-SG7-ThurkC, color tan, by restricting the superimposition to the cyclase domains. In all of these models except EryKC, there is a conserved D/E/N residue in at least one position of the triad.

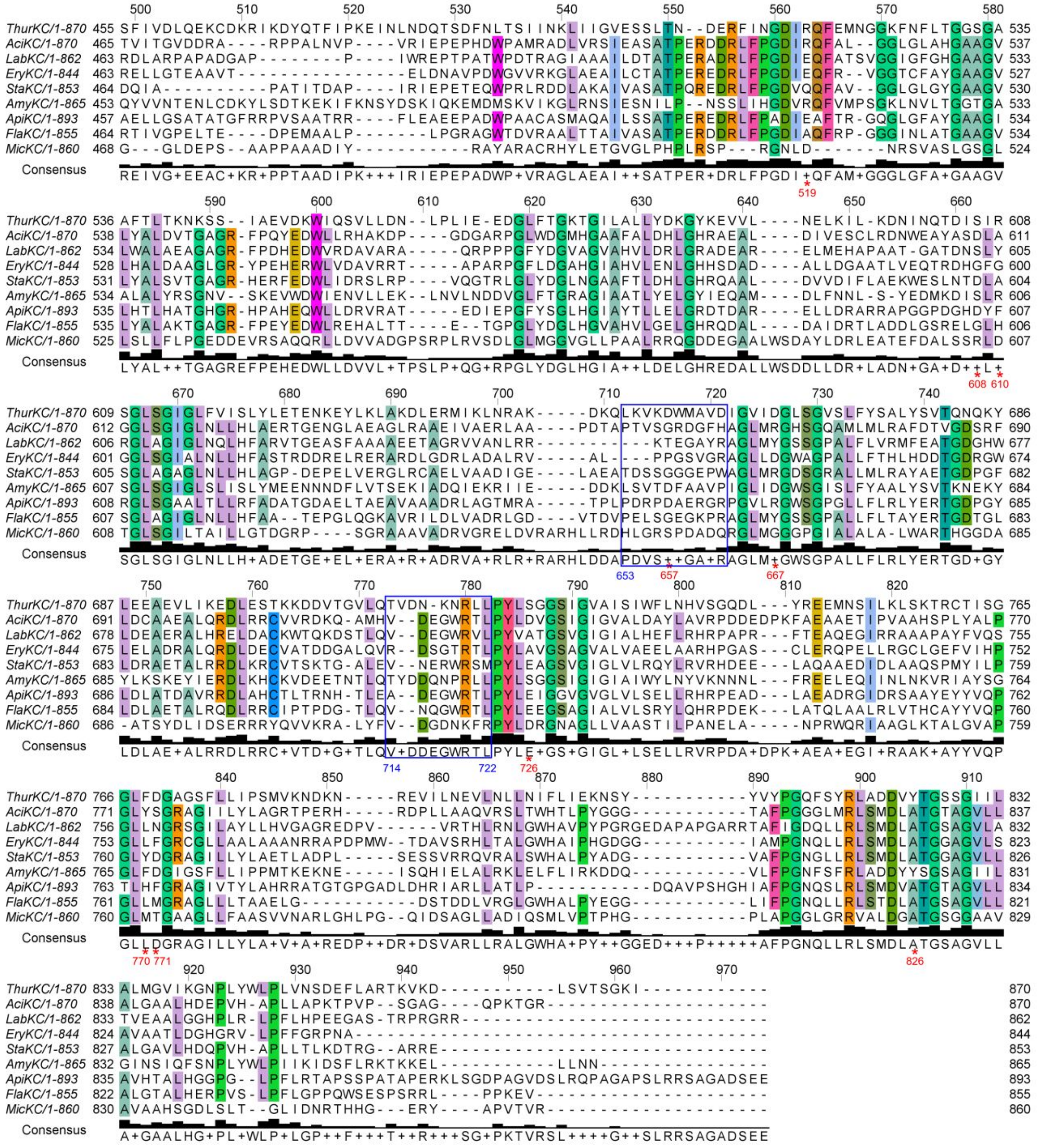

Figure S17: Sequence alignment of cyclase domains of class III LanKC reported to produce lanthipeptide containing labionin. The alignment was performed by the Clustal Omega tool using the order of input. Above 50% conserved in sequence alignment is highlited and image is created using Jalview software. Conserved residues predicted to be relevant to catalytic activity are marked with an asterisk and corresponding residue number in ThurKC sequence. Loops that are predicted to orient the ThurA$_1$ leader and referred to in the main text are marked with an blue box and labeled with corresponding residue number in the ThurKC sequence.

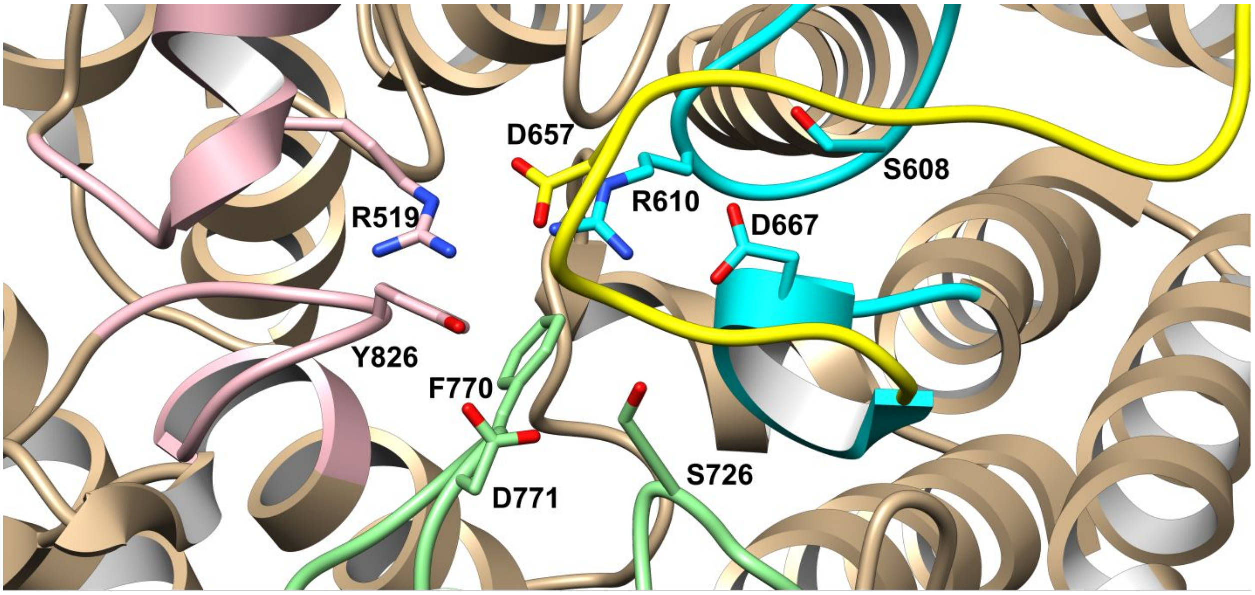

ThurKC	AciKC	LabKC	EryKC	StaKC	AmyKC	ApiKC	FlaKC	MicKC	% conserved in loop region
S608	***D609***	**S603**	***H597***	***D602***	**S604**	***D605***	G604	***R605***	25
R610	A611	**R606**	G600	A604	**R608**	F607	**H606**	***D607***	100
D667	***R669***	Y656	**D653**	***R661***	**D663**	***R664***	Y662	G665	75
F770	**Y773**	L758	L755	**Y762**	**F767**	H765	L763	**M762**	88
D771	***S774***	**N759**	***F756***	**D763**	**D768**	***F776***	***M764***	***T763***	87.5
S726	***D726***	T715	V711	E718	**S723**	***E721***	***E719***	***D720***	38
Y826	A829	T825	A815	A818	**Y823**	A826	L763	A821	12.5
R519	**R521**	E515	E511	Q514	**R515**	E517	A517	**H503**	37.5
D657	**D661**	**E647**	P643	**D648**	**D659**	**D654**	**E649**	**D659**	88

Figure S18: Alphafold predictions suggest conservation of crucial residues in the cyclase domain loop region of labionin producing LanKCs. A sequence alignment for the cyclase domains of LanKC enzymes reported to produce labionin rings did not reflect conservation of residues potentially important for catalysis. Superimpositions of Alphafold predictions of these enzymes show that although the residues may not be directly superimposable, they are present in the nearby loop region in the cyclase active site cavity. The 4 loop regions are color coded. Residues directly superimposing the original ThurKC residues in other Class III Lanthionine synthetases are to their right. If the residue is conserved, it is bolded. If the residue is conserved somewhere else in that loop region, it is bold and italics.

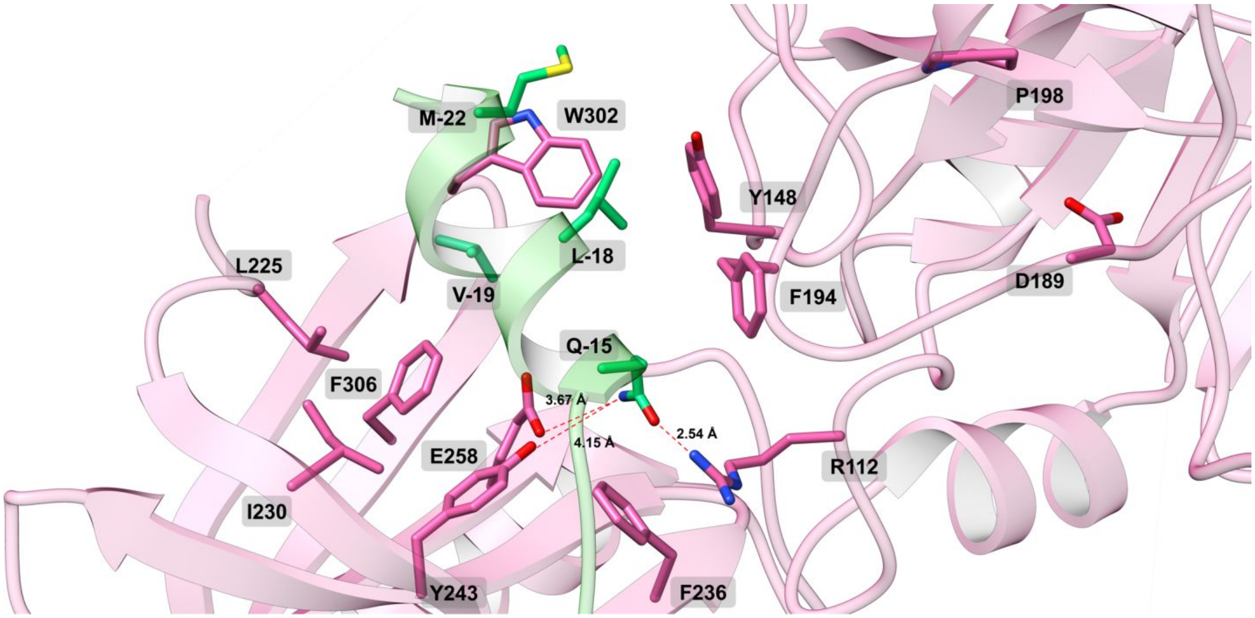

Figure S19: Leader binding region of LP-SG7-ThurKC. $ThurA_1$ leader peptide ranging from Asp(-10) to Met(-22) is shown as a green ribbon and amino acid side chains are shown as sticks, and the residues in the kinase and lyase domains interacting with them are in pink.

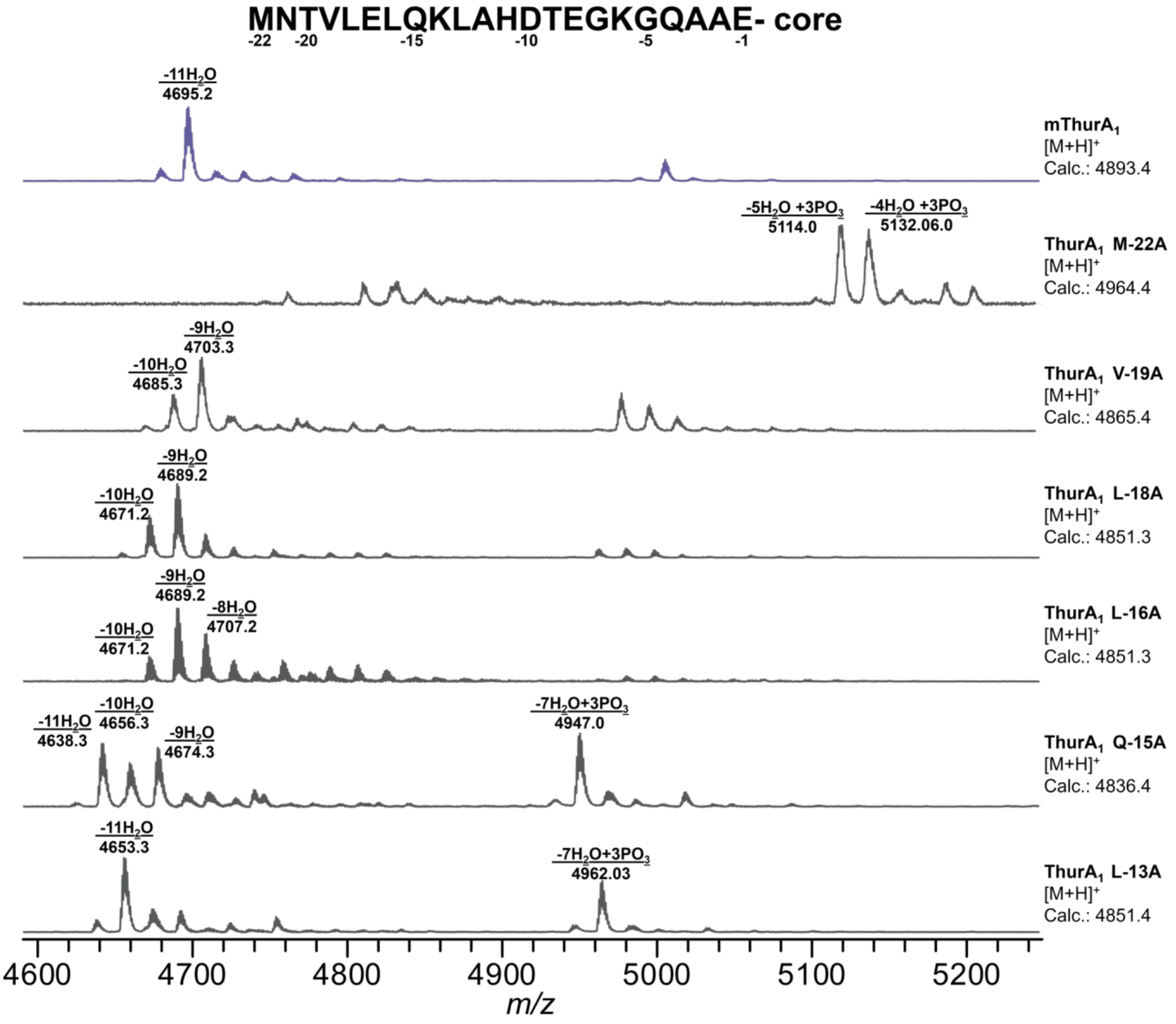

Figure S20: Heterologous co-expression of ThurA$_1$ peptide leader mutants with WT ThurKC in *E. coli* and analysis by MALDI-TOF MS.

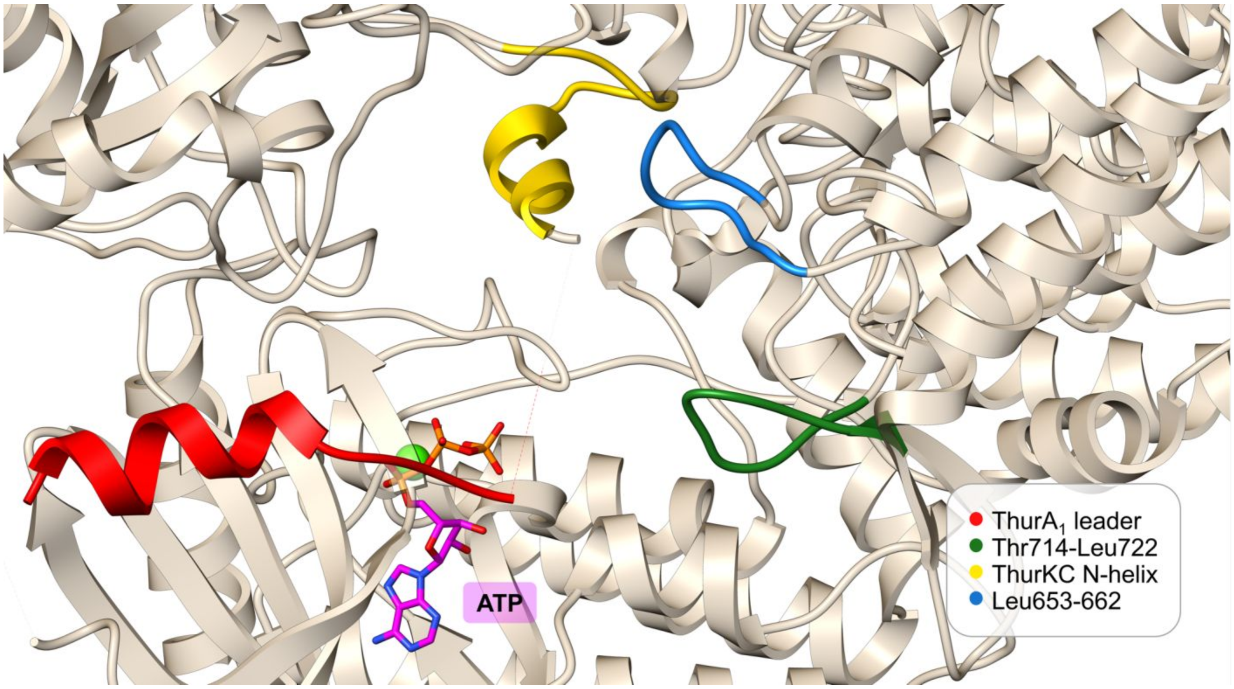

Figure S21: Basis for orientation of the core peptide. Structural features from each domain may help direct the core peptide towards the active site(s) and away from solvent.

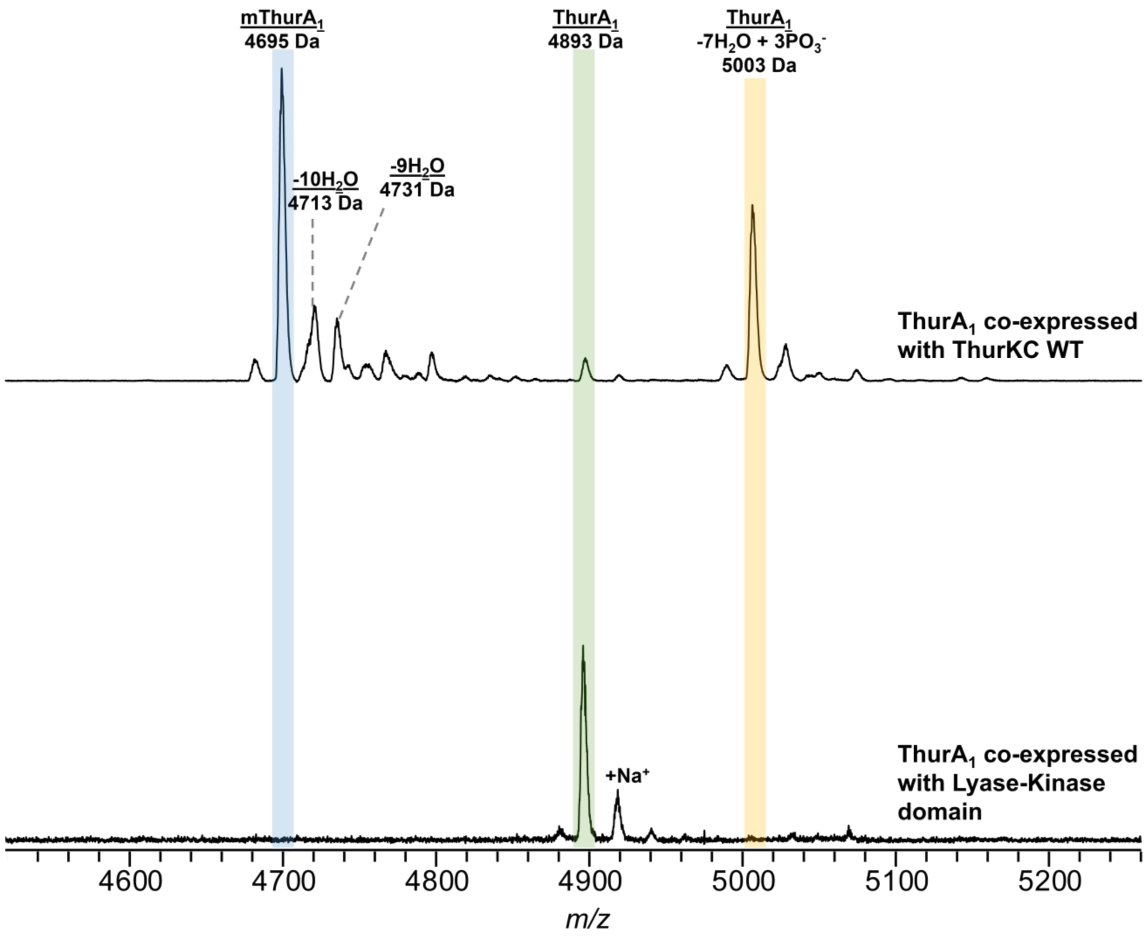

Figure S22: MALDI-TOF MS analysis of $ThurA_1$ precursor peptide co-expressed with wild-type and truncated ThurKC. Wild-type ThurKC can effectively modify the full-length precursor peptide to produce $mThurA_1$ with 11 dehydrations, but co-expression of the full-length precursor with the Lyase-Kinase did not result in any modifications.

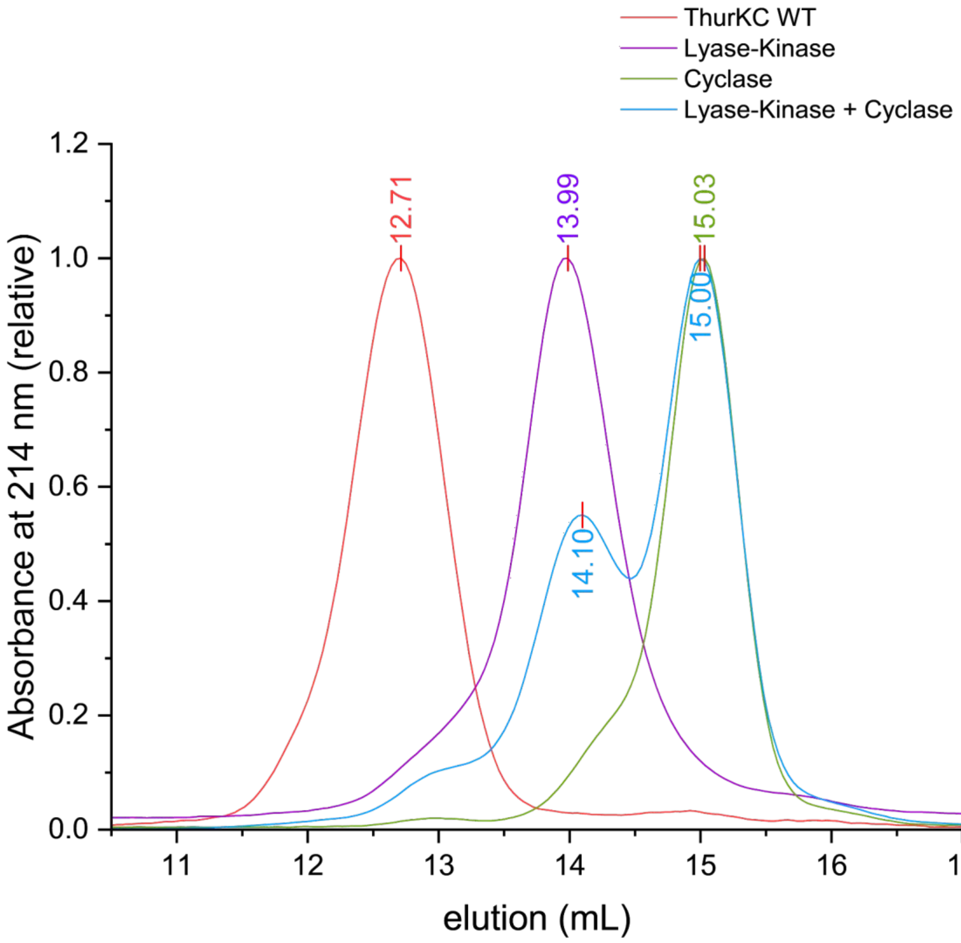

Figure S23: Analytical Size Exclusion Chromatography of ThurKC and its subdomains.
No complex is observed when the Lyase and Kinase domain are incubated with the cyclase domain and run in SEC, with the retention time marked above or below each peak in the corresponding color. The calculated MW of $NHis_6$ WT ThurKC is 101.8 kDa, $NHis_6$ lyase-kinase domain is 59.0 kDa, and the cyclase domain is 43.2 kDa. For reference, the size exclusion standards elute at the following elution volumes: Blue Dextran (MW 2,000 kDa) at 8.41 mL, β-Amylase from sweet potato at (MW 200 kDa) at 11.22 mL, Alcohol Dehydrogenase from yeast, (MW 150 kDa) at 12.09 mL, Albumin from bovine serum (MW 66 kDa) elutes at 13.38 mL, Carbonic Anhydrase from bovine erythrocytes (MW 29 kDa) at 15.863 mL, and Cytochrome C from horse heart at (MW 12.4 kD) at 17.179 mL.

References

(1) Walden, H., Selenium incorporation using recombinant techniques. *Acta Crystallogr D Biol Crystallogr* **2010,** *66*, 352-7.

(2) Xue, D.; Older, E. A.; Zhong, Z.; Shang, Z.; Chen, N.; Dittenhauser, N.; Hou, L.; Cai, P.; Walla, M. D.; Dong, S. H., et al., Correlational networking guides the discovery of unclustered lanthipeptide protease-encoding genes. *Nat Commun* **2022,** *13*, 1647.

(3) Meyer, H. E.; Heber, M.; Eisermann, B.; Korte, H.; Metzger, J. W.; Jung, G., Sequence analysis of lantibiotics: chemical derivatization procedures allow a fast access to complete Edman degradation. *Anal Biochem* **1994,** *223*, 185-90.

(4) Vonrhein, C.; Flensburg, C.; Keller, P.; Sharff, A.; Smart, O.; Paciorek, W.; Womack, T.; Bricogne, G., Data processing and analysis with the autoPROC toolbox. *Acta Crystallogr D Biol Crystallogr* **2011,** *67*, 293-302.

(5) Pannu, N. S.; Waterreus, W. J.; Skubak, P.; Sikharulidze, I.; Abrahams, J. P.; de Graaff, R. A., Recent advances in the CRANK software suite for experimental phasing. *Acta Crystallogr D Biol Crystallogr* **2011,** *67*, 331-7.

(6) Winn, M. D.; Ballard, C. C.; Cowtan, K. D.; Dodson, E. J.; Emsley, P.; Evans, P. R.; Keegan, R. M.; Krissinel, E. B.; Leslie, A. G.; McCoy, A., et al., Overview of the CCP4 suite and current developments. *Acta Crystallogr D Biol Crystallogr* **2011,** *67*, 235-42.

(7) Bond, P. S.; Cowtan, K. D., ModelCraft: an advanced automated model-building pipeline using Buccaneer. *Acta Crystallogr D Struct Biol* **2022,** *78*, 1090-1098.

(8) Emsley, P.; Cowtan, K., Coot: model-building tools for molecular graphics. *Acta Crystallogr D Biol Crystallogr* **2004,** *60*, 2126-32.

(9) Oeffner, R. D.; Bunkoczi, G.; McCoy, A. J.; Read, R. J., Improved estimates of coordinate error for molecular replacement. *Acta Crystallogr D Biol Crystallogr* **2013,** *69*, 2209-15.

(10) Murshudov, G. N.; Skubak, P.; Lebedev, A. A.; Pannu, N. S.; Steiner, R. A.; Nicholls, R. A.; Winn, M. D.; Long, F.; Vagin, A. A., REFMAC5 for the refinement of macromolecular crystal structures. *Acta Crystallogr D Biol Crystallogr* **2011,** *67*, 355-67.

(11) Afonine, P. V.; Grosse-Kunstleve, R. W.; Echols, N.; Headd, J. J.; Moriarty, N. W.; Mustyakimov, M.; Terwilliger, T. C.; Urzhumtsev, A.; Zwart, P. H.; Adams, P. D., Towards automated crystallographic structure refinement with phenix.refine. *Acta Crystallogr D Biol Crystallogr* **2012,** *68*, 352-67.

(12) Ongpipattanakul, C.; Liu, S.; Luo, Y.; Nair, S. K.; van der Donk, W. A., The mechanism of thia-Michael addition catalyzed by LanC enzymes. *Proc Natl Acad Sci U S A* **2023,** *120*, e2217523120.
